# RTS,S/AS01E Malaria Vaccine Induces Memory and Polyfunctional T Cell Responses in a Pediatric African Phase III Trial

**DOI:** 10.3389/fimmu.2017.01008

**Published:** 2017-08-23

**Authors:** Gemma Moncunill, Stephen C. De Rosa, Aintzane Ayestaran, Augusto J. Nhabomba, Maximillian Mpina, Kristen W. Cohen, Chenjerai Jairoce, Tobias Rutishauser, Joseph J. Campo, Jaroslaw Harezlak, Héctor Sanz, Núria Díez-Padrisa, Nana Aba Williams, Daryl Morris, John J. Aponte, Clarissa Valim, Claudia Daubenberger, Carlota Dobaño, M. Juliana McElrath

**Affiliations:** ^1^ISGlobal, Barcelona Ctr. Int. Health Res. (CRESIB), Hospital Clínic – Universitat de Barcelona, Barcelona, Spain; ^2^Vaccine and Infectious Disease Division, Fred Hutchinson Cancer Research Center, Seattle, WA, United States; ^3^Centro de Investigação em Saúde de Manhiça (CISM), Maputo, Mozambique; ^4^Department of Laboratory Medicine, University of Washington, Seattle, WA, United States; ^5^Ifakara Health Institute, Bagamoyo Research and Training Centre, Bagamoyo, Tanzania; ^6^Swiss Tropical and Public Health Institute, Basel, Switzerland; ^7^University of Basel, Basel, Switzerland; ^8^Department of Epidemiology and Biostatistics, School of Public Health-Bloomington, Indiana University, Bloomington, IN, United States; ^9^Department of Osteopathic Medical Specialties, Michigan State University, East Lansing, MI, United States; ^10^Department of Immunology and Infectious Diseases, Harvard T.H. Chen School of Public Health, Boston, MA, United States; ^11^Department of Medicine, University of Washington, Seattle, WA, United States

**Keywords:** malaria, *Plasmodium falciparum*, vaccine, cellular immune responses, T cells, intracellular cytokine staining, flow cytometry

## Abstract

Comprehensive assessment of cellular responses to the RTS,S/AS01E vaccine is needed to understand potential correlates and ultimately mechanisms of protection against malaria disease. Cellular responses recognizing the RTS,S/AS01E-containing circumsporozoite protein (CSP) and Hepatitis B surface antigen (HBsAg) were assessed before and 1 month after primary vaccination by intracellular cytokine staining and 16-color flow cytometry in 105 RTS,S/AS01-vaccinated and 74 rabies-vaccinated participants (controls) in a pediatric phase III trial in Africa. RTS,S/AS01E-vaccinated children had significantly higher frequencies of CSP- and HBsAg-specific CD4^+^ T cells producing IL-2, TNF-α, and CD40L and HBsAg-specific CD4^+^ T producing IFN-γ and IL-17 than baseline and the control group. Vaccine-induced responses were identified in both central and effector memory (EM) compartments. EM CD4^+^ T cells expressing IL-4 and IL-21 were detected recognizing both vaccine antigens. Consistently higher response rates to both antigens in RTS,S/AS01E-vaccinated than comparator-vaccinated children were observed. RTS,S/AS01E induced polyfunctional CSP- and HBsAg-specific CD4^+^ T cells, with a greater degree of polyfunctionality in HBsAg responses. In conclusion, RTS,S/AS01E vaccine induces T cells of higher functional heterogeneity and polyfunctionality than previously characterized. Responses detected in memory CD4^+^ T cell compartments may provide correlates of RTS,S/AS01-induced immunity and duration of protection in future correlates of immunity studies.

## Introduction

A highly efficacious vaccine can substantially contribute to control and eventual elimination of malaria. This life-threatening disease caused an estimated 429,000 deaths in 2015 (1), mainly among sub-Saharan African children. In 2009–2014, the RTS,S/AS01E malaria vaccine was evaluated in a pediatric Phase III trial in Africa (2–4). Vaccine efficacy (VE) against clinical malaria over 1 year post-immunization was moderate (56%) in children enrolled at age 5–17 months and low (31%) in infants enrolled at age 6–12 weeks. Importantly, the protective effects waned quickly over time. To better understand why the RTS,S/AS01E vaccine induced only partial and short-lived protection against malaria, a thorough examination of the immune responses elicited, including different effector functions and memory phenotypes, using qualified or validated assays to ensure appropriate assay sensitivity and specificity to detect small frequencies of antigen-specific cells is needed.

RTS,S is a vaccine based on the circumsporozoite protein (CSP) of *Plasmodium falciparum*, targeting the sporozoite and liver stages of infection. This self-assembling virus-like particle consists of a recombinant protein containing part of CSP fused to the hepatitis B surface antigen (HBsAg) and it is coexpressed with HBsAg alone. In the Phase III trial, RTS,S was formulated with AS01E liposomal adjuvant containing monophosphoryl lipid A and QS21 and was designed to induce strong anti-CSP antibody and T helper (T_H_) 1 cell responses. Accordingly, in past clinical trials in endemic areas, RTS,S consistently induced high anti-CSP IgG titers (5–11) and moderate T_H_1 CD4^+^ T cell responses (5–10). IgG titers have been recently shown to correlate with vaccine-induced protection in secondary analysis of Phase III trial data (12, 13). However, IgG responses do not explain why RTS,S/AS01E VE is moderate or short-lived.

Regarding the cellular responses, CD4^+^ T cells expressing IL-2, TNF-α, and IFN-γ (and CD40L in naïve adults) have been detected by intracellular cytokine staining (ICS) or ELISpot (5–11, 14, 15) upon vaccination. In vaccinated naïve adults challenged with *P. falciparum*-infected mosquitoes, CSP-specific CD4^+^ T cells and IFN-γ measured by ELISPot were associated with protection (11, 14, 15). One study evaluating RTS,S/AS02D in African children less than 1 year old did not find any association with protection (5), whereas in another one with RTS,S/AS01E in children 5–17 months old observed CSP-specific TNF-α^+^ CD4^+^ T cell responses to be associated with a reduced risk of clinical malaria independently of anti-CSP IgG titers (8). Polyfunctional analysis of the ICS data of the later study showed that IFN-γ^-^IL-2^−^TNF-α^+^ CD4^+^ T cells independently predicted reduced risk of clinical malaria, although the response was also detected in control vaccinees, and found a synergistic interaction with anti-CSP IgG titers (9). CD8^+^ T cell responses were only detected in humans in two studies, one in infants in whom the responses were not correlated with protection and the other in naïve adults (5, 14). Interestingly, NK cells were found to be the main producers of IFN-γ in one field trial, but its association with protection was not assessed (10). Overall, no clear cellular correlates of protection have been demonstrated in African trials although only a limited number of assays restricted to a few immune variables have been studied. There has been no assessment of other cell effector functions, such as T_H_2 or follicular helper T cells (T_FH_), or memory phenotypes that may be induced by RTS,S/AS01E and could be correlated with antibody responses and involved in vaccine-induced protection. Interestingly, in the Phase III trial, we detected T_H_1 responses in supernatants of CSP-stimulated cells associated with protection in RTS,S/AS01E vaccinees, whereas a T_H_2 cytokine, IL-5, was associated with increased risk for malaria (16). To our knowledge, T_H_2 responses had only been examined in one previous study in humans, where IL-4 was found elevated in culture supernatants from RTS,S/AS02D-vaccinated infants (5). Lastly, memory T cell subsets have only been examined in a study with malaria-naïve adults who underwent a challenge with *P. falciparum*-infected mosquitoes after RTS,S vaccination. In that study, central memory T (T_CM_) cells and effector/effector memory T cells from RTS,S-vaccinated adults produced IL-2 after *ex vivo* CSP stimulation and frequencies were higher in protected vs. non-protected subjects (15).

Assessing the memory phenotype, the polyfunctionality degree and other relevant functions besides T_H_1 responses, such as T_H_2, T_H_17, cytotoxic, or immunoregulatory responses, may be key to identify functionally complex responses to RTS,S/AS01E and unravel its mode of action. In fact, complexity of the immune response to malaria and the partial and short-lived protection induced by RTS,S/AS01E stresses the need to expand the breadth of immunological profiling to T_H_2- and regulatory-type markers. This may be particularly relevant in infants in African settings, as they are exposed to prenatal and environmental factors that may modulate immune response to vaccines.

The aim of this study was to analyze RTS,S/AS01E cellular immunogenicity after primary vaccination using two qualified 16-color multiparametric ICS assays that allow the assessment of memory cell subsets and regulatory, cytotoxic, T_H_1, T_H_2, T_H_17, T_FH_ effector functions, most of them never assessed before, and to identify and establish a baseline of cell phenotypes and functional responses to be evaluated in studies of immune correlates of protection elicited by the vaccine. To this end, we analyzed the CSP- and HBsAg-specific cells *ex vivo* using previously cryopreserved peripheral blood mononuclear cells (PBMC) isolated from a subset of children aged 5–17 months at enrollment from Tanzania and Mozambique and following receipt of either the RTS,S/AS01E vaccine or a comparator rabies vaccine.

## Materials and Methods

### Study Population and Study Design

We performed a study on a subset of 179 children aged 5–17 months from the RTS,S/AS01E Phase III trial (ClinicalTrials.gov NCT00866619), described elsewhere (4): 105 children received RTS,S/AS01E and 74 children received the rabies vaccine as a comparator at study months zero (M0), one, and two. PBMC were collected at M0 before vaccination and approximately 30 days after the third vaccination dose (M3). RTS,S/AS01E-vaccinated and rabies-vaccinated children were randomly selected for this study among participants with no reported malaria episodes defined by observation of *P. falciparum* parasites on blood smears, identified through passive case detection during 18 months of follow-up after third vaccination dose. Of note, PBMC samples from children who had malaria cases were reserved for future correlates analyses to test the selected markers identified in this study. Samples were collected in two different African centers: Manhiça Health Research Center, Fundação Manhiça (FM-CISM, Mozambique; 120 children), and Ifakara Health Institute and Bagamoyo Research and Training Centre (IHI-BRTC, Tanzania; 59 children). The two sites had low-medium malaria transmission intensity at the time of the study (2–4). Investigators conducted all assays blinded to vaccination status.

### Sample Collection

Blood was collected in 5 ml sodium citrate (BD Vacutainer^®^ CPT™) tubes. PBMCs were isolated by density gradient centrifugation, cryopreserved and shipped to the Fred Hutchinson Cancer Research Center where the PBMC were thawed and stained (see Methods in Supplementary Material).

### PBMC Stimulations

Thawed PBMC were rested in a 37°C, 5% CO_2_ incubator overnight. The resting step increases the sensitivity of the assay (data not shown), probably by decreasing the stress and activation of PBMC due to the thawing process and exposure to the toxic cryopreservation agent. PBMC were stimulated with peptide pools covering the HBsAg or the CSP antigen present in the RTS,S vaccine (Table S1 in Supplementary Material). Negative controls contained 0.5% DMSO, the diluent for peptide pools, and Staphylococcal enterotoxin B was used as positive control stimulation at 1 µg/ml. Cultures were incubated 6 h at 37°C, 5% CO_2_. This short incubation time increases the sensitivity and specificity of the assay to detect antigen-specific cells, avoiding non-specific and secondary immune responses. Further details are found in Supplementary Material.

### Intracellular Cytokine Staining

Peripheral blood mononuclear cells were stained with one of two 16-color ICS panels that were designed for the study and that had previously undergone assay qualification with a formerly validated panel (17, 18). Cell staining was performed as described (17) (Supplementary Material). Antibody details can be found in Tables S2 and S3 in Supplementary Material. Data were acquired using a BD LSR II flow cytometer (BD Biosciences) directly from the plates using a high throughput sampler. We found some toxicity of HBsAg peptides, but since we could exclude dead cells, data were considered acceptable for analysis. We noted some spillover from CD154 to CXCR5 and therefore, T_FH_-like cells were excluded from the analysis. Flow cytometry analysis was performed using FlowJo software (Version 9.9 Tree Star). Gating strategies for both panels are shown in Figures S1 and S2 in Supplementary Material.

### Statistical Analysis

The raw FCS files and manual gates were imported into the R environment (19) using the OpenCyto framework (20) and cell counts for the cell gates of interest were obtained for all stimulations and subjects.

For the analysis of the effect of vaccination on the frequencies of cells expressing the functional markers, a multivariate linear mixed effect model was fitted using logarithm-10 transformed cell frequencies (cells expressing the functional marker/total number of cells within each cell subset) as an outcome. These models are commonly used (21–24) and allow to model jointly the different cell stimulations that each subject sample underwent using random effects. The model included a random intercept for the subject effect nested within stimulation and a random slope for timepoint. In these models, observations generated by different subjects (biological replicates) are assumed to be independent. We included the following predictors in the regression models (as fixed effects): stimulation (CSP, DMSO, or HBsAg and DMSO, as appropriate), time of visit (pre- vs. post-vaccination), and vaccination status (RTS,S/AS01E vs. comparator vaccine), as well as all interactions between the three factors to allow variations of responses across subgroups of vaccinees and time. Linear combination of coefficients in these models allowed addressing specific study questions (details in Supplementary Material). Through these models, we estimated the % change in cell frequencies in RTS,S/AS01E vs. comparator vaccinees at each timepoint, the % change from M0 to M3 in each vaccine group, and the % change in RTS,S/AS01E vs. comparator vaccinees accounting for both timepoints. Statistical significance of comparisons was based on likelihood ratio tests. Antigen-specific responses were analyzed accounting for background, i.e., DMSO stimulation.

To define positivity of responses in each cell subset and subject, we identified the functional markers that were differentially expressed between the antigen (CSP or HBsAg) stimulations and their corresponding background (DMSO control stimulation) for each cell subset, subject, and at each timepoint. Thus, the proportion of cells expressing that marker was compared between the two stimulation conditions for each subject and timepoint. The statistical method used was mixture models for single-cell assays (MIMOSA) (25), using the default algorithm (Expectation–Maximization algorithm) and a false-discovery rate of 0.05%. This method was chosen because it has higher sensitivity and specificity than alternative methods such as Fisher’s exact test (25). A separate model for each functional marker, cell subset, and stimulation was fitted. Once we had defined the positivity or negativity for each functional marker, cell subset, subject, and timepoint, then the proportion of children with positive responses between vaccine groups at baseline or 1-month post-vaccination was compared using two-sided Fisher’s exact test.

Polyfunctional responses (cell subsets that express multiple functional markers) were analyzed using combinatorial polyfunctionality analysis of antigen-specific T cell subsets (COMPASS) (26). COMPASS models all cell subsets expressing functional markers simultaneously and selects the subsets most likely to have a positive antigen-specific response. The antigen-specific response is quantified by the probability of having a positive response. COMPASS functionality and polyfunctionality scores, summarizing each subject’s polyfunctional profile, were compared between vaccine groups using Wilcoxon test. The functionality score is the estimated proportion of antigen-specific cell subsets detected among all possible ones, whereas the polyfunctionality score is similar but weighted by the degree of polyfunctionality. In addition, COMPASS generates heatmaps that show the posterior probabilities for each modeled cell subset for each subject. CD107a marker was eliminated from MIMOSA and COMPASS analysis, because peptide pool stimulation was associated with a non-specific increase in CD107a compared to background, and response positivity could not be defined.

Statistical tests were considered significant at 0.05 α-level. Adjustments for multiple testing were done using Holm (27) and Benjamini–Hochberg (28) approaches. All analyses were conducted using R software. See Supplementary Material for detailed descriptions.

## Results

### Study Population

We used two different multiparameter ICS panels to assess cellular immune responses specific to the RTS,S/AS01E vaccine antigens. PBMC from 55 RTS,S and 41 comparator vaccinees, and 50 RTS,S and 33 comparator vaccinees were stained with antibody panel 1 (P1) and panel 2 (P2), respectively. The two panels had the same set of core markers, which allowed the exclusion of dead cells, monocytes (CD14), identification of CD4^+^ and CD8^+^ T cells (CD4, CD8 and CD3), NK and NK-T cells (CD56), the T_H_1 cytokines IFN-γ, IL-2, and TNF-α, the T_H_2 cytokine IL-4, the costimulatory molecule CD154 (CD40L), and the cytotoxic marker granzyme B (Figures S1 and S2 in Supplementary Material; Tables S2 and S3 in Supplementary Material). P1 additionally had CD45RA and CCR7 markers that allowed the identification of memory cell subsets, and IL-21, a cytokine related to T_FH_. Panel 2 (P2) included γδ TCR for the identification of γδ T cells and several functional markers: the regulatory cytokine IL-10, the T_H_2 cytokine IL-13, the T_H_17 cytokine IL-17, and cytotoxicity marker CD107a. The gating strategy is detailed in Figures S1 and S2 in Supplementary Material. The common set of core markers (lineage and functional markers) allowed the pooled analysis for these markers. Males and females were similarly represented in both study sites and vaccine groups, with a proportion of 59 and 56.8% females in the RTS,S/AS01E and comparator groups, respectively.

### Frequencies of CSP- and HBsAg-Specific CD4^+^ T Cells Induced by RTS,S/AS01E

**Figure 1:**
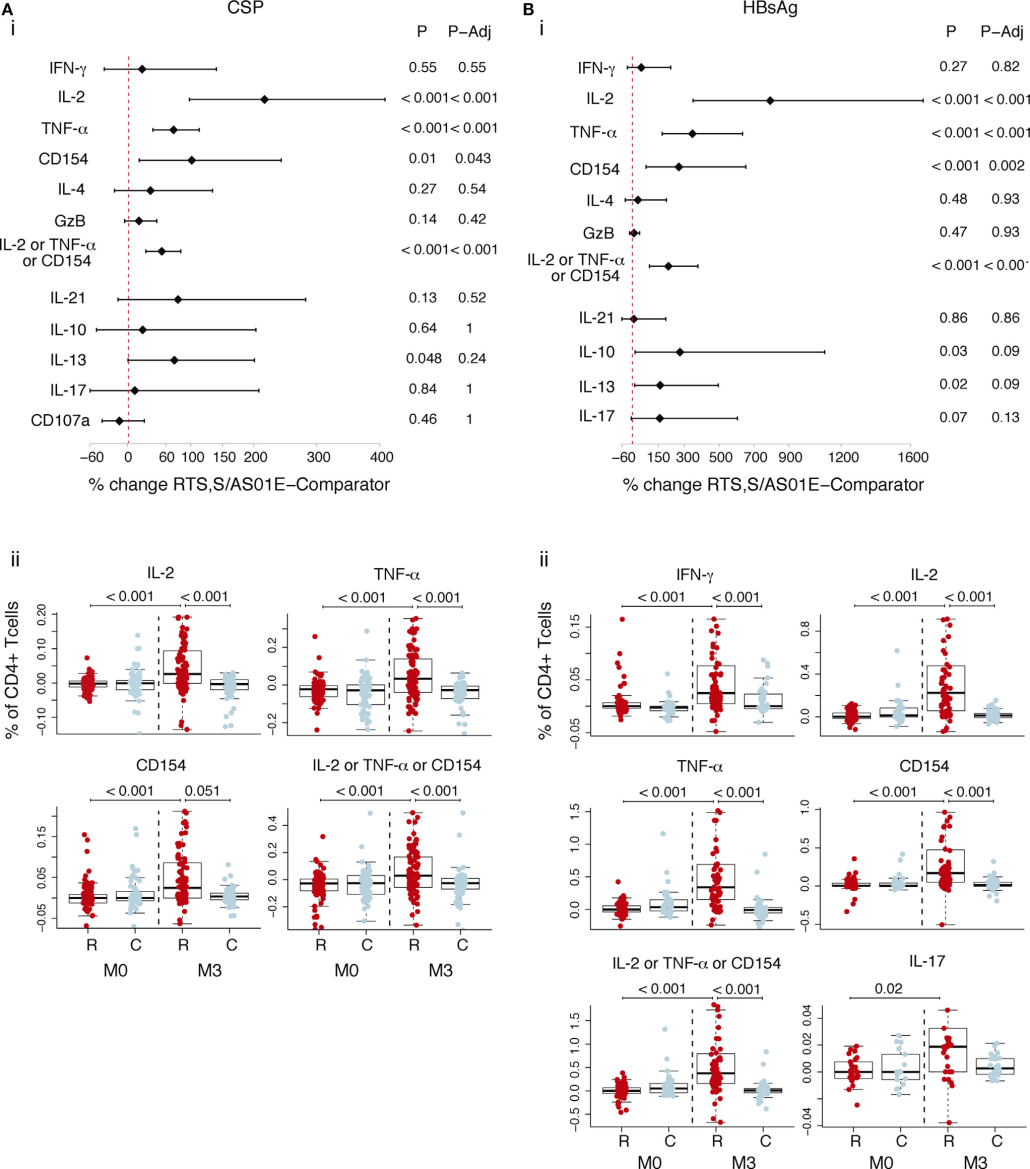
CSP- and hepatitis B surface antigen (HBsAg)-specific CD4^+^ T cell responses induced by RTS,S/AS01E. CSP- **(A)** and HBsAg- **(B)** specific CD4^+^ T cells expressing the common functional markers of both intracellular cytokine staining panels. (i) Forest plot showing the overall effect of RTS,S/AS01E (R) vaccination from baseline (M0) to 1 month post-third immunization (M3) taking into account the M0–M3 changes in comparator (C) vaccinees. The % change between RTS,S/AS01E and comparator vaccinees taking into account M0–M3 changes and 95% confidence intervals shown were obtained with a multivariate linear mixed effect model. *P* values (P) were obtained through likelihood ratio test and were adjusted for multiple testing (P-Adj) through Holm’s approach. (ii) Box plots showing the frequencies of CD4^+^ T cells expressing the functional markers after background subtraction found to be statistically significant in (i). Boxplots illustrate the medians and the 25th and 75th quartiles, and whiskers display 1.5 times interquartile ranges, outliers are not shown to facilitate visualization of the differences between comparison groups. Differences between vaccine groups at M0 and at M3 and differences from M0 to M3 within each vaccine group were computed through a multivariate linear mixed effect model and *P* values obtained through likelihood ratio test and were adjusted for multiple testing through Holm approach. Only significant *P* values adjusted for multiple testing are shown. Sample size in **(A)**, for markers detected by both staining panels *N* = 100 RTS,S/AS01E and 65 comparator at M0, 100 RTS,S/AS01E and 70 comparator at M3. For comparisons including M0 and M3, only subjects that had samples at both timepoints are included (*N* = 156 for markers detected by both panels, *N* = 83 for markers detected by panel 1, *N* = 73 for markers detected by panel 2). Sample size in **(B)** *N* = 62 RTS,S/AS01E and 36 comparator at M0, 67 RTS,S/AS01E and 50 comparator at M3. For IL-17 (detected by panel 2) *N* = 30 RTS,S/AS01E and 18 comparator at M0, 37 RTS,S/AS01E and 27 comparator at M3. For comparisons including M0 and M3, only subjects that had samples at both timepoints are included (*N* = 71 for markers detected by both panels, *N* = 35 for markers detected by panel 1, *N* = 36 for markers detected by panel 2).

We compared frequencies of CSP- or HBsAg-specific T cells expressing functional markers in RTS,S- and rabies-vaccinated children, accounting for the pre-vaccination frequencies in a multivariate model. RTS,S/AS01E-vaccinated children had a statistically significant increase of CSP-specific CD4^+^ T cells expressing IL-2 (217% increase), TNF-α (72.3%), and CD154 (101.4%) from baseline to post-vaccination in contrast to the rabies vaccinees (Figure 1A, i). We observed larger increases over time of HBsAg-specific CD4^+^ T cells expressing IL-2 (732.2%), TNF-α (346%), and CD154 (268.3%) (Figure 1B, i) in RTS,S/AS01E than in rabies vaccinees.

Figure 1A (ii) and Figure 1B (ii) show the frequencies of CSP- and HBsAg-specific CD4^+^ T cells after background subtraction. Higher frequencies of CSP- and HBsAg-specific CD4^+^ T cells expressing the above markers, and additionally HBsAg-specific CD4^+^ T cells expressing IFN-γ and IL-17 cells, were observed in RTS,S/AS01E vaccinees at post-vaccination compared to baseline and to comparators. No differences were detected between the two vaccine groups at baseline, or from baseline to post-vaccination in comparator vaccinees, the latter suggesting that there was no effect of naturally acquired immunity on cellular responses.

**Figure 2:**
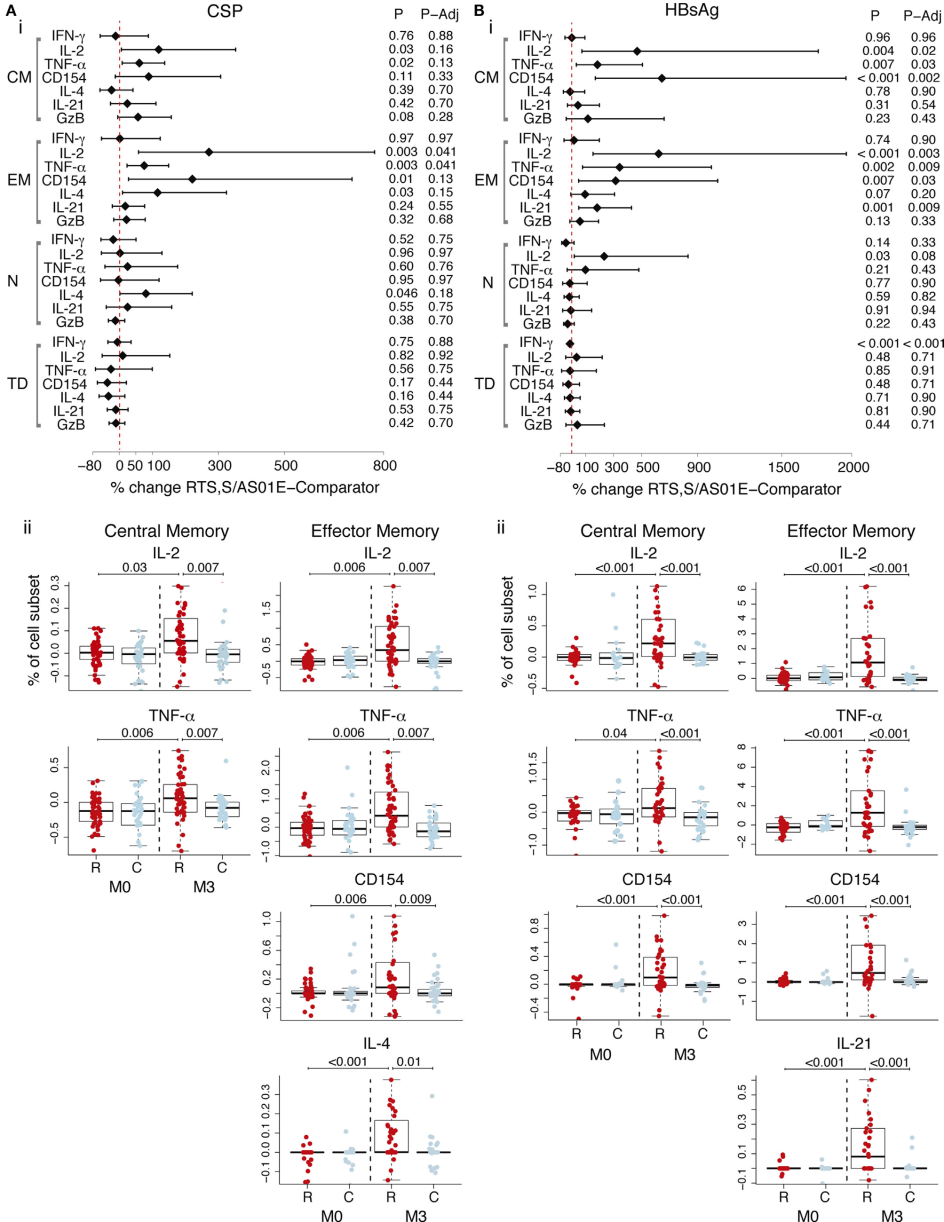
CSP- and hepatitis B surface antigen (HBsAg)-specific CD4^+^ T cell memory responses induced by RTS,S/AS01E. CSP- **(A)** and HBsAg- **(B)** specific memory CD4^+^ T cell subsets [central memory (CM); effector memory (EM); naive (N); terminally differentiated (TD)] expressing functional markers measured in the intracellular cytokine staining panels panel 1. (i) Forest plot showing the overall effect of RTS,S/AS01E (R) vaccination from baseline (M0) to 1 month post-third immunization (M3) taking into account the M0–M3 changes in comparator (C) vaccinees. The % change between RTS,S/AS01E and comparator vaccinees and 95% confidence intervals shown were obtained with a multivariate linear mixed effect model. *P* values (P) were obtained through likelihood ratio test and were adjusted for multiple testing (P-Adj) through Benjamini–Hochberg approach. (ii) Box plots showing the frequencies of CD4^+^ T_CM_ and CD4^+^ T_EM_ cells expressing selected functional markers after background subtraction. Boxplots illustrate the medians and the 25th and 75th quartiles, and whiskers display 1.5 times interquartile ranges, outliers are not shown to facilitate visualization of the differences between comparison groups. Differences between vaccine groups at M0 and at M3 and differences from M0 to M3 within each vaccine group were computed through a multivariate linear mixed effect model and *P* values obtained through likelihood ratio test and were adjusted for multiple testing through Holm approach. Only significant *P* values adjusted for multiple testing are shown. Sample size in **(A)** For *N* = 53 RTS,S/AS01E and 35 comparator at M0, 52 RTS,S/AS01E and 39 comparator at M3. For comparisons including M0 and M3, only subjects that had samples at both timepoints are included (*N* = 83). Sample size in **(B)** *N* = 32 RTS,S/AS01E and 18 comparator at M0, 37 RTS,S/AS01E and 27 comparator at M3. For comparisons including M0 and M3, only subjects that had samples at both timepoints are included (*N* = 35).

Antigen-specific T cells in memory subsets, defined by CD45RA and CCR7, were analyzed following vaccination. CD4^+^ T_CM_ (CD45RA^−^ CCR7^+^) and CD4^+^ T_EM_ (CD45RA^−^ CCR7^−^) cells recognized both vaccine antigens. After accounting for baseline frequencies, RTS,S/AS01E vaccinees had more CSP-specific CD4^+^ T_EM_ cells producing IL-2 and TNF-α, respectively, than comparator vaccinees (Figure 2A, i). Comparisons between vaccine groups at post-vaccination and from baseline to post-vaccination revealed additional responses: higher frequencies of CD4^+^ T_CM_ expressing IL-2 and TNF-α and CD4^+^ T_EM_ cells expressing CD154, IL-4 and IL-21, in RTS,S/AS01E than comparator vaccinees (Figure 2A, ii). Regarding HBsAg-specific responses, RTS,S/AS01E vaccinees had increased frequencies of CD4^+^ T_CM_ producing IL-2, TNF-α and CD154 and CD4^+^ T_EM_ producing IL-2, TNF-α, CD154 and IL-21 than comparator vaccinees (Figure 2B, i).

Therefore, RTS,S/AS01E vaccination induced CSP and HBsAg-specific CD4^+^ T_CM_ and CD4^+^ T_EM_ with T_H_1, and additional T_H_2, and T_FH_ functions. No overall effect of RTS,S vaccination was detected on the frequencies of terminally differentiated CD4^+^ T cells (CD45RA^+^ CCR7^−^), naïve CD4^+^ T cells (CD45RA^+^ CCR7^+^), other analyzed cell subsets (NK, NK-T like, γδ T, CD8^+^ T cells) or on other functional markers (Tables S5 and S6 in Supplementary Material).

### Non-Specific CD4^+^ T Cell and CD8^+^ T Cell Responses Induced by RTS,S/AS01E Vaccination

**Figure 3:**
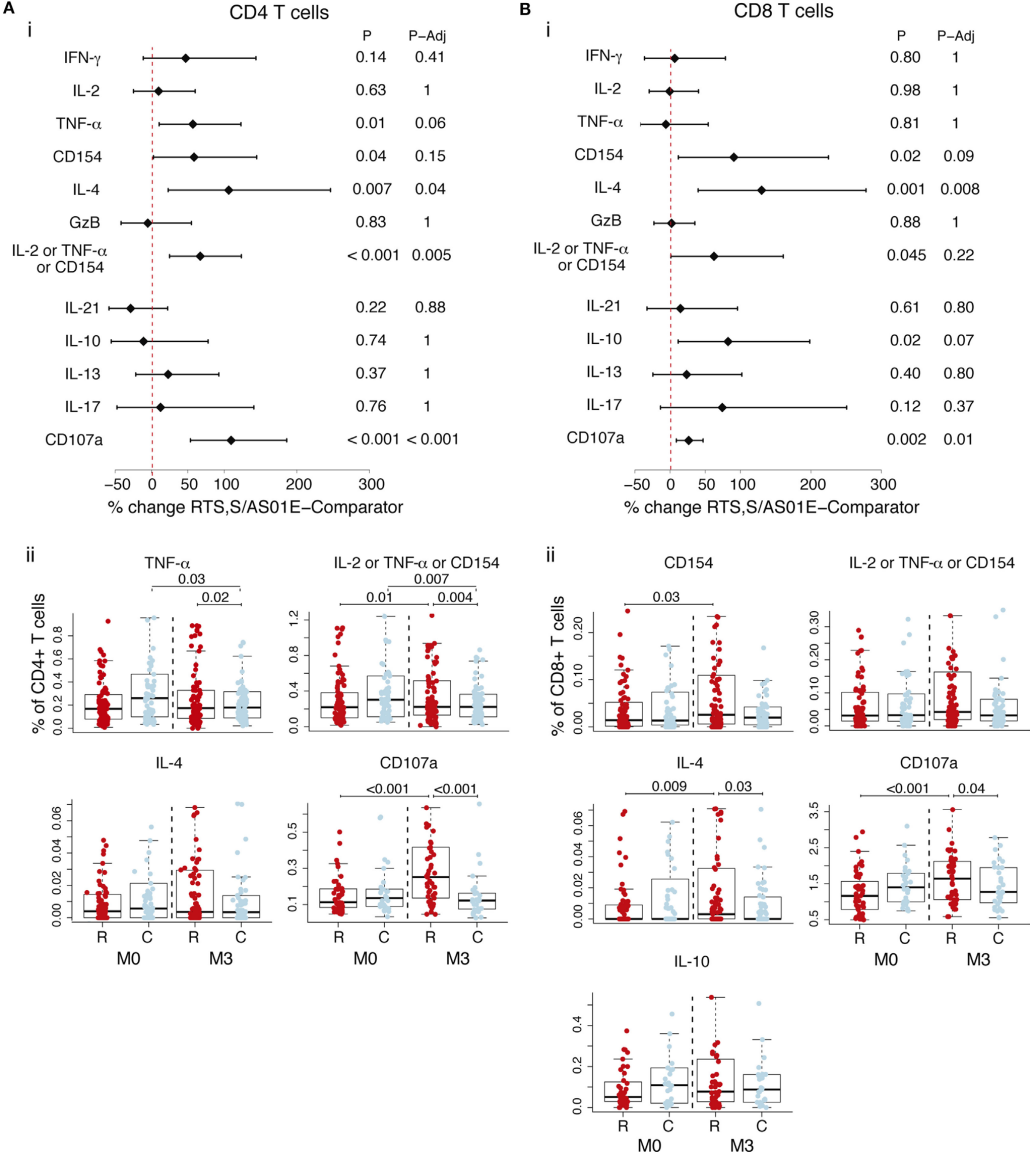
Non-specific CD4^+^ T and CD8^+^ T cell responses induced by RTS,S/AS01E as determined by the difference between M0 and M3 for the control stimulation. Frequencies in control stimulations (stimulation with the peptide diluent, DMSO, considered background) of CD4^+^ T cells **(A)** and CD8^+^ T cells **(B)** expressing the common functional markers of both intracellular cytokine staining panels and also functional markers measured separately in each panel. (i) Forest plot showing the overall effect of RTS,S/AS01E (R) vaccination from baseline (M0) to 1 month post-third immunization (M3) taking into account the M0–M3 changes in comparator (C) vaccinees. The % change between RTS,S/AS01E and comparator vaccinees and 95% confidence intervals shown were obtained with a multivariate linear mixed effect model. *P* values (P) were obtained through likelihood ratio test and were adjusted for multiple testing (P-Adj) through Holm approach. (ii) Box plots showing the frequencies of T cells expressing functional markers when significant differences were detected. Boxplots illustrate the medians and the 25th and 75th quartiles, and whiskers display 1.5 times interquartile ranges, outliers are not shown to facilitate visualization of the differences between comparison groups. Differences between vaccine groups at M0 and at M3 and differences from M0 to M3 within each vaccine group were computed through a multivariate linear mixed effect model and *P* values obtained through likelihood ratio test and were adjusted for multiple testing through Holm approach. Only significant *P* values adjusted for multiple testing are shown. Sample size in **(A)** for markers detected by both staining panels *N* = 100 RTS,S/AS01E and 65 comparator at M0, 101 RTS,S/AS01E and 70 comparator at M3. For CD107a (detected by panel 2), *N* = 47 RTS,S/AS01E and 30 comparator at M0, 49 RTS,S/AS01E and 31 comparator at M3. For comparisons, including M0 and M3, only subjects that had samples at both timepoints are included (*N* = 157 for markers detected by both panels, *N* = 83 for markers detected by panel 1 and *N* = 74 for markers detected by panel 2). Sample size in **(B)** for markers common from both staining panels *N* = 100 RTS,S/AS01E and 64 comparator at M0, 99 RTS,S/AS01E and 69 comparator at M3. For CD107a *N* = 47 RTS,S/AS01E and 30 comparator at M0, 47 RTS,S/AS01E and 31 comparator at M3. For comparisons including M0 and M3, only subjects that had samples at both timepoints are included (*N* = 154 for markers detected by both panels, *N* = 82 for markers detected by panel 1 and *N* = 742 for markers detected by panel 2).

Analysis of frequencies of cell subsets expressing functional markers in the background (DMSO, serving as the unstimulated control) revealed non-specific responses (i.e., not specific for CSP or HBsAg) upon RTS,S/AS01E vaccination (Figure 3). Accounting for baseline and responses in comparator vaccinees, RTS,S/AS01E vaccination increased the frequencies of CD4^+^ T cells producing IL-2 or TNF-α or CD154, IL-4, and CD107a (Figure 3A, i). In comparator vaccinees, a decrease in these two subsets from baseline to post-vaccination was detected. Interestingly, RTS,S/AS01E vaccination also increased the frequencies of CD8^+^ T cells producing IL-4 and CD107a (Figure 3B, i).

Since we detected an effect of RTS,S/AS01E vaccination in the background, we looked at the *ex vivo* antigen stimulations when not accounting for background. RTS,S/AS01E-vaccinated children had CD4^+^ T cells and CD8^+^ T cells expressing IL-4 and CD107a, as was observed for the background alone and, thus, likely reflecting non-specific responses (Figure S3 in Supplementary Material). Additionally, RTS,S/AS01E vaccination increased the frequencies of CD4^+^ T cells expressing IL-13 and CD8^+^ T cells expressing CD154 and IL-2 or TNF-α or CD154 following CSP stimulation when not taking background into account (Figure S3 in Supplementary Material). These additional responses in RTS,S/AS01E-vaccinated children were no longer significant when taking into account the background and likely reflect a non-specific effect of RTS,S/AS01E vaccination.

### Higher Proportion of Positive CD4^+^ T Cell Responses Defined by MIMOSA in RTS,S/AS01E than Comparators

To assess the vaccine specificity of the antigen-specific T cell responses detected and the rate of responders to the RTS,S/AS01E vaccine (participants whose T-cells responded to stimulation), we determined the positivity of the responses for each subject comparing the frequencies of T cells expressing a functional marker in the antigen-stimulated and unstimulated control conditions for each participant using the MIMOSA statistical method. If a response is truly vaccine specific, one would expect to detect it after vaccination but not at baseline or in the comparator vaccinees. We found a significantly higher proportion of IL-2^+^, TNF-α^+^, and CD154^+^ CD4^+^ T cell positive CSP-specific responses in RTS,S/AS01E than in comparator vaccinees post-vaccination (Table 1). Response rates for these markers ranged from 24 to 30% in RTS,S/AS01E vaccinees; whereas for comparator vaccinees, there were none or very few responses (Table 1). Similarly, CSP-specific positive responses were absent or very low at baseline, supporting the specificity of the definition of positivity (Table S7 in Supplementary Material).

**Table 1:** Comparison of proportion of positive CD4^+^ T cell responses to CSP and hepatitis B surface antigen (HBsAg) between RTS,S/AS01E and comparator vaccinees 1-month post-third vaccination.

		CSP				HBsAg			
		RTS,S/AS01E	Comparator	*P*-value		RTS,S/AS01E	Comparator	*P*-value	
			
Functional marker^a^	Intracellular cytokine staining panel^b^	Positive/total (%)	Positive/total (%)	Raw *P*^c^	Adj *P*^d^	Positive/total (%)	Positive/total (%)	Raw *P*^c^	Adj *P*^d^
IFN-γ	P1 and P2	1/100 (1%)	0/70 (0%)	1	1	**34/67 (50.75%)**	**9/50 (18%)**	**<0.001**	**0.001**
IL-2	P1 and P2	**30/100 (30%)**	**0/70 (0%)**	**<0.001**	**<0.001**	**49/67 (73.13%)**	**9/50 (18%)**	**<0.001**	**<0.001**
TNF-α	P1 and P2	**24/100 (24%)**	**0/70 (0%)**	**<0.001**	**<0.001**	**48/67 (71.64%)**	**4/50 (8%)**	**<0.001**	**<0.001**
CD154	P1 and P2	**29/100 (29%)**	**5/70 (7.14%)**	**<0.001**	**0.002**	**51/67 (76.12%)**	**15/50 (30%)**	**<0.001**	**<0.001**
IL-4	P1 and P2	0/100 (0%)	2/70 (2.86%)	0.17	0.5	17/67 (25.37%)	6/50 (12%)	0.1	0.2
IL-2 or TNF-α or CD154	P1 and P2	**21/100 (21%)**	**0/70 (0%)**	**<0.001**	**<0.001**	**46/67 (68.66%)**	**2/50 (4%)**	**<0.001**	**<0.001**
IL-21	P1	2/52 (3.85%)	0/39 (0%)	0.5	1	20/37 (54.05%)	12/27 (44.44%)	0.61	1
IL-10	P2	0/48 (0%)	0/31 (0%)	1	1	3/30 (10%)	2/23 (8.7%)	1	1
IL-13	P2	5/48 (10.42%)	1/31 (3.23%)	0.39	1	**20/30 (66.67%)**	**8/23 (34.78%)**	**0.03**	0.11
IL-17	P2	1/48 (2.08%)	0/31 (0%)	1	1	**8/30 (26.67%)**	**0/23 (0%)**	**0.007**	**0.03**

*Bold font indicates differences with p-values <0.05*. *^a^Granzyme B was included in the analysis, but not shown because it is constitutively expressed independently of cell stimulation and activation*.

*^b^Data from markers detected by both staining panels, P1 and P2, are combined*.

*^c^Raw P, original P-value computed based on two-sided Fisher’s exact test*.

*^d^Adj P, P values adjusted for multiple testing through Holm’s approach*.

For HBsAg, we additionally detected more IFN-γ^+^ and IL-17^+^ CD4^+^ T cell positive responses in RTS,S/AS01E than comparator vaccinees at post-vaccination. Rates of HBsAg responders ranged from 26.7 to 76.1% in RTS,S/AS01E vaccinees (Table 1). However, a proportion of comparator vaccinees at post-vaccination and all vaccinees at M0 were also positive (Table 1; Table S7 in Supplementary Material), likely due to the fact that these children received the hepatitis B vaccine during the Expanded Program of Immunization (EPI) before the study. Most of the CSP responders were also HBsAg responders (Figure S4 in Supplementary Material).

When looking at CD4^+^ T_CM_ and CD4^+^ T_EM_ cell subsets, we found a significantly higher proportion of CSP responders in RTS,S/AS01E than in comparator vaccinees for IL-2, TNF-α, and CD154 (Table 2). Regarding HBsAg responses, RTS,S/AS01E vaccinees had higher rates of IL-2 and CD154 responses in CD4^+^ T_CM_ cells and IFN-γ, IL-2, TNF-α, CD154 and IL-21 responses in CD4^+^ T_EM_ than comparator vaccinees. Very few positive responses to CSP and HBsAg were detected in comparator vaccinees or at baseline (Table 2; Table S8 in Supplementary Material). Almost no positive responses were found in the other cell subsets analyzed (CD4^+^ T_TD_, CD4^+^ T_N_, NK, NK-T like, γδ T, CD8^+^ T cells, and memory CD8^+^ T cell subsets), and no significant differences were detected between vaccination groups for any of the two vaccine antigens (Tables S9–S11 in Supplementary Material).

**Table 2:** Comparison of proportion of positive responses to CSP and hepatitis B surface antigen (HBsAg) in memory CD4^+^ T cell subsets between RTS,S/AS01E and comparator vaccinees 1-month post-third vaccination.

		CSP				HBsAg			
		RTS,S/AS01E	Comparator	*P*-value		RTS,S/AS01E	Comparator	*P*-value	
			
Memory subset	Functional marker^a^	Positive/total (%)	Positive/total (%)	Raw *P*^b^	Adj *P*^c^	Positive/total (%)	Positive/total (%)	Raw *P*^b^	Adj *P*^c^
CM	IFN-γ	1/52 (1.92%)	0/39 (0%)	1	1	1/37 (2.7%)	0/27 (0%)	1	1
	IL-2	**12/52 (23.08%)**	**0/39 (0%)**	**<0.001**	**0.009**	**14/37 (37.84%)**	**0/27 (0%)**	**<0.001**	**0.001**
	TNF-α	**8/52 (15.38%)**	**0/39 (0%)**	**0.01**	**0.049**	**8/37 (21.62%)**	**0/27 (0%)**	**0.02**	0.058
	CD154	**11/52 (21.15%)**	**0/39 (0%)**	**0.002**	**0.01**	**12/37 (32.43%)**	**1/27 (3.7%)**	**0.005**	**0.02**
	IL-4	0/52 (0%)	0/39 (0%)	1	1	2/37 (5.41%)	0/27 (0%)	0.5	1
	IL-21	0/52 (0%)	0/39 (0%)	1	1	0/37 (0%)	0/27 (0%)	1	1
EM	IFN-γ	1/52 (1.92%)	0/39 (0%)	1	1	**9/37 (24.32%)**	**0/27 (0%)**	**0.008**	**0.03**
	IL-2	**21/52 (40.38%)**	**0/39 (0%)**	**<0.001**	**<0.001**	**23/37 (62.16%)**	**1/27 (3.7%)**	**<0.001**	**<0.001**
	TNF-α	**12/52 (23.08%)**	**0/39 (0%)**	**<0.001**	**0.009**	**18/37 (48.65%)**	**0/27 (0%)**	**<0.001**	**<0.001**
	CD154	**14/52 (26.92%)**	**2/39 (5.13%)**	**0.01**	**0.049**	**21/37 (56.76%)**	**1/27 (3.7%)**	**<0.001**	**<0.001**
	IL-4	4/52 (7.69%)	0/39 (0%)	0.13	0.46	8/37 (21.62%)	1/27 (3.7%)	0.07	0.19
	IL-21	**7/52 (13.46%)**	**0/39 (0%)**	**0.02**	0.07	**16/37 (43.24%)**	**2/27 (7.41%)**	**0.002**	**0.01**
TD	IFN-γ	0/52 (0%)	0/39 (0%)	1	1	0/37 (0%)	0/27 (0%)	1	1
	IL-2	0/52 (0%)	0/39 (0%)	1	1	0/37 (0%)	0/27 (0%)	1	1
	TNF-α	0/52 (0%)	0/39 (0%)	1	1	0/37 (0%)	0/27 (0%)	1	1
	CD154	0/52 (0%)	0/39 (0%)	1	1	0/37 (0%)	0/27 (0%)	1	1
	IL-4	0/52 (0%)	0/39 (0%)	1	1	0/37 (0%)	0/27 (0%)	1	1
	IL-21	0/52 (0%)	0/39 (0%)	1	1	0/37 (0%)	0/27 (0%)	1	1
Naive	IFN-γ	0/52 (0%)	0/39 (0%)	1	1	1/37 (2.7%)	2/27 (7.41%)	0.57	1
	IL-2	0/52 (0%)	0/39 (0%)	1	1	4/37 (10.81%)	2/27 (7.41%)	1	1
	TNF-α	0/52 (0%)	0/39 (0%)	1	1	5/37 (13.51%)	0/27 (0%)	0.07	0.19
	CD154	0/52 (0%)	0/39 (0%)	1	1	1/37 (2.7%)	0/27 (0%)	1	1
	IL-4	0/52 (0%)	0/39 (0%)	1	1	0/37 (0%)	0/27 (0%)	1	1
	IL-21	0/52 (0%)	0/39 (0%)	1	1	14/37 (37.84%)	11/27 (40.74%)	1	1

*Bold font indicates differences with p-values <0.05*.

*CM, central memory (CD45RA^−^ CCR7^+^); EM, effector memory (CD45RA^−^ CCR7^−^); TD, terminally differentiated (CD45RA^+^ CCR7^−^)*.

*^a^Granzyme B was included in the analysis, but not shown because it is constitutively expressed independently of cell stimulation and activation*.

*^b^Raw P, original P-value computed based on two-sided Fisher’s exact test*.

*^c^Adj P, P values adjusted for multiple testing through Benjamini–Hochberg approach*.

### CSP- and HBsAg-Specific Polyfunctional CD4^+^ T Cell Responses Induced by RTS,S/AS01E

**Figure 4:**
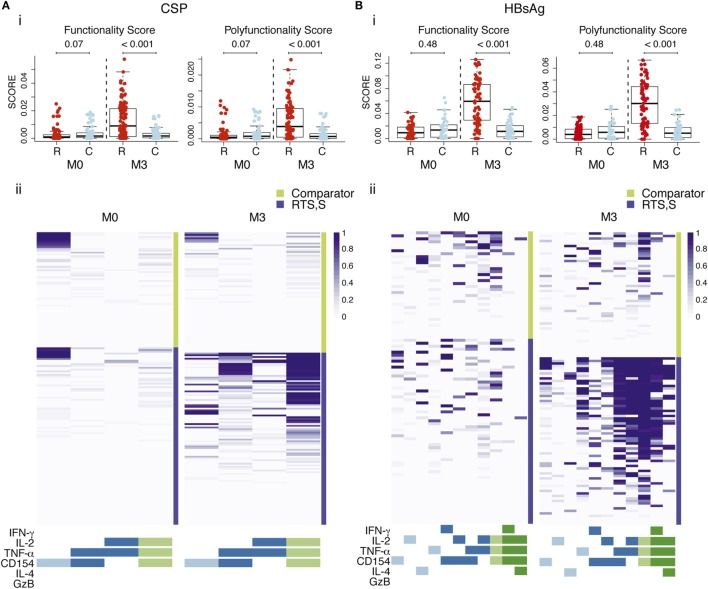
CSP- **(A)** and hepatitis B surface antigen (HBsAg)-specific **(B)** CD4^+^ T cell polyfunctional responses. (i) Box plots of functionality and polyfunctionality scores calculated by combinatorial polyfunctionality analysis of antigen-specific T cell subsets (COMPASS), stratified by vaccine group and timepoint. The functionality score represents the estimated proportion of cell subsets showing positive responses among all possible cell subsets expressing any functional marker, whereas the polyfunctional score is similar but it is weighted by the degree of polyfunctionality. Boxplots illustrate the medians and the 25th and 75th quartiles, and whiskers display 1.5 times interquartile ranges. COMPASS analysis was performed using the common markers of both panels and all subjects with data (IL-4, IFN-γ, granzyme B (GzB), IL-2, TNF-α, CD154, 64 possible subsets). *P* values computed through Wilcoxon tests and adjusted for multiple testing through Holm approach separately for CSP and HBsAg antigens are shown. (ii) Heatmap of COMPASS posterior probabilities showing CD4^+^ T cell responses to CSP and HBsAg in RTS,S/AS01E and comparator vaccinees at pre-vaccination (M0) and 1 month post-third vaccination (M3). Columns represent functional cell subsets with detectable antigen-specific responses, color coded by the number of functional markers they express and are ordered by increasing degree of polyfunctionality. Rows represent study children, which are stratified by vaccine status, at the top the comparator vaccinees and at the bottom the RTS,S/AS01E vaccinees. Each cell shows the probability (color coded by purple intensity) that the corresponding child shows an antigen-specific response in the corresponding cell subset. Sample size in **(A)** *N* = 100 RTS,S/AS01E and 65 comparator at M0, 100 RTS,S/AS01E and 70 comparator at M3; **(B)** *N* = 62 RTS,S/AS01E and 36 comparator at M0, 67 RTS,S/AS01E and 50 comparator at M3.

When analyzing the pooled data for the functional markers included in both panels, RTS,S/AS01 vaccinees had significantly higher functionality and polyfunctionality scores for CSP- and HBsAg-specific CD4^+^ T cells than comparator vaccinees (Figures 4A,B). Heatmaps of posterior probabilities of responses showed CSP-specific CD4^+^ T cell responses among subsets coexpressing three markers (IL-2, TNF-α, and CD154), and two markers (TNF-α and CD154; IL-2 and TNF-α) in RTS,S/AS01E vaccinees, whereas almost no responses were detected in comparator vaccinees or at baseline (Figure 4A, ii). For HBsAg responses, more CD4^+^ T cell subsets with positive responses and with higher degree of polyfunctionality were detected in RTS,S/AS01E vaccinees (Figure 4B, ii). Besides the same triple- and double-positive CD4^+^ T cell responses recognizing CSP antigen, we also detected CD154^+^ IL-2^+^ TNF-α^+^ IFN-γ^+^ CD4^+^ T cells and CD154^+^ IL-2^+^ TNF-α^+^ IL4^+^ CD4^+^ T cells. By contrast, comparator vaccinees or all vaccinees at baseline had none or weak responses in these subsets that could be explained by background responses to previous malaria exposure or hepatitis B vaccination. Therefore, RTS,S/AS01E induced polyfunctional CD4^+^ T cells to both vaccine antigens, with a higher degree of polyfunctionality for HBsAg that included T_H_1 and T_H_2 responses.

When the COMPASS analysis was performed using all the functional makers included in each antibody panel separately, we identified additional highly polyfunctional CD4^+^ T cell subsets coexpressing IL-13 (for both vaccine antigens) or IL-21 (for HBsAg) in addition to IL-2, TNF-α and CD154 (Figure S5 in Supplementary Material). This further highlights the induction of highly polyfunctional CD4^+^ T cell subsets with a T_H_2 and T_FH_ functions by RTS,S/AS01E. No polyfunctional responses were detected in CD8^+^ T cells for either vaccine antigen.

## Discussion

We provide a detailed characterization of the *ex vivo* antigen-specific T cell response induced by RTS,S/AS01E vaccination in a pediatric Phase III trial (2–4). In addition to previously described IL-2- and TNF-α-expressing CD4^+^ T cell responses (5–10), we have identified for the first time in a pediatric field trial T_H_2 effector functions and IL-21 in RTS,S vaccinees, attributed the responses to the central memory (CM) and effector memory (EM) compartments, and observed polyfunctional CD4^+^ T cell responses, which may be a key feature of a protective response. Although this analysis was limited to vaccine recipients who did not become infected with *P. falciparum*, this study has identified key immune responses that can be examined in a larger case-control study within this trial.

RTS,S/AS01E vaccination induced distinct antigen-specific CD4^+^ T cell populations in a subset of children: CSP-specific IL-2, TNF-α, and CD154 CD4^+^ T cell responses and HBsAg-specific IFN-γ, IL-2, TNF-α, CD154, and IL-17 CD4^+^ T cell responses. Frequencies of cytokine-positive CD4^+^ T cells were consistent with the frequencies of IL-2 and TNF-α CSP-specific responses reported previously in RTS,S Phase II trials in endemic areas (5–10). Although no clear associations of TNF-α and IL-2 responses with protection were found in past field studies, they could contribute in RTS,S-induced protection (8, 9). TNF-α is an effector cytokine that may mediate mechanisms of *P. falciparum* pre-erythrocytic protection (29–31) and IL-2 induces proliferation of T cells and amplifies effector functions. CD4^+^ T cells producing IL-2 may contribute to the memory pool of CD4^+^ T cells with effector functions since they can be maintained during long periods of time and can develop into IFN-γ-producing T cells following subsequent stimulation (32). Of note, rates of IL-2 CSP responders (23–40% depending on the CD4^+^ T cell subset) were similar to the estimated VE in the Phase III trial (50% after a year of follow-up or 28% till study end) (4). IL-2 expression can also induce NK activation and secretion of IFN-γ (10, 33, 34), a key effector cytokine involved in malaria protection (35). Contrary to previous findings, we did not detect IFN-γ expression in CD4^+^ T cells or NK cells *ex vivo* following CSP stimulation (5, 7, 8, 10, 14). This is probably due to the short stimulation time in our study compared to longer times in whole blood assays from previous studies, which allowed bystander activation of CD4^+^ T cells and NK cells through IL-2 (10). Remarkably, we detected CD40L expression in antigen-specific CD4^+^ T cells, in contrast to previous field studies (6, 7).

Importantly, CD4^+^ T cell responses were detected in CM and EM compartments. Memory phenotypes are relevant to define high-quality and long-lasting responses, for instance, the higher proliferative potential of CM cells has been associated with protection from infection (32, 36–38). CD4^+^ T_CM_ responses could, therefore, be involved in long-lasting protection, whereas the CD4^+^ T_EM_ responses could be associated with the short protection observed in the trial. IL2^+^, CD154^+^, and TNF-α^+^ CD4^+^ T_CM_ cells were detected, whereas CD4^+^ T_EM_ cells had additional effector functions, in accordance with the different cytokine profiles associated with these memory T cell subsets (32, 39, 40) and studies reporting CD4^+^ T_EM_ cells as the main producers of effector cytokines (32, 40). Higher frequencies of CD4^+^ T_EM_ cells expressing IL-4, and IL-21 for both vaccine antigens, and IFN-γ for HBsAg, were detected in RTS,S/AS01E-vaccinated children compared to baseline frequencies or comparator vaccinees. T_H_2 (IL-4) and T_FH_ (IL-21) effector functions together with CD40L could provide help to B cells for humoral responses and may correlate with antibody responses (41–44). No responses were detected in CD4^+^ T_TD_ cells, a cell subset with high IFN-γ effector function, with no long-term memory potential and fated for death (32).

RTS,S/AS01E vaccination induced polyfunctional CSP- and HBsAg-specific CD4^+^ T cells. This finding is particularly important since multifunctional T cells have been associated with higher quality responses and risk (26) or protection from infections (45, 46). Most CSP and HBsAg positive responses were found in the triple functional marker CD4^+^ T cell subset coexpressing IL-2, TNF-α, and CD154, although we also observed responses in a 4-function subset coexpressing IL-13. For HBsAg, we also found numerous responses in CD4^+^ T cells subsets simultaneously producing four functional markers: IL-2, TNF-α, CD154 plus IL-21 or IFN-γ or IL-4, reflecting T_FH_, T_H_1 and T_H_2 distinct differentiation of these effector cell subsets.

Overall, HBsAg-specific responses were of higher magnitude, effector breadth and polyfunctionality than CSP-specific responses, suggesting a higher quality of response probably due to a booster effect since children should have been previously vaccinated with hepatitis B, or a higher immunogenicity of HBsAg compared to CSP due to the higher proportion of HBsAg than CSP in RTS,S (4:1).

RTS,S/AS01E may have a non-specific effect since higher background CD4^+^ T cell and CD8^+^ T cell responses were detected after vaccination compared with baseline. Since these responses included IL-4 and CD107a, it could be indicating a bias to a T_H_2 status and higher cytolytic potential of T cells in RTS,S/AS01E-vaccinated children. There is increasing evidence of non-specific effect of vaccines (47, 48), but a non-specific effect of RTS,S/AS01E may still have an impact on the response against *P. falciparum* infection. The significance of these responses may be worth noting in the context of the future correlates analyses. This finding of a non-specific effect highlights the importance of correcting for the background (by subtracting background) in order to assess antigen-specific effects.

In this study, we only included children without experiencing a documented malaria episode during the 18 months of follow-up; therefore, the clinical relevance of our findings will be assessed in future correlates studies. The exclusion of malaria cases is unlikely to bias results and affect study conclusions since malaria transmission intensity was low at that time in Manhiça and Bagamoyo and lack of malaria is not likely to be due solely to vaccine-induced protection, but also to lack of exposure to the mosquito-bearing parasite. Another potential limitation of our study relates to the toxicity of HBsAg peptide concentration used. Although this could impact cellular responsiveness, we did exclude dead and dying cells in the analysis, and it is likely that any impact would affect both vaccine groups.

The breadth of functions, patterns, and variability of responses to CSP, together with memory phenotypes of responding cell subsets described in our study, reflects a complex response to the RTS,S/AS01 vaccine. These responses, together with anti-CSP IgG data, may provide insights into the lack of protection in a substantial proportion of vaccinees, and may be key in providing correlates for VE and duration of protection.

## Ethics Statement

Approval for the study protocol was obtained from the Ethical Committee of the Hospital Clínic in Barcelona (CEIC, Spain), the National Health and Bioethics Committee (CNBS, Mozambique), the Ethikkommission Beider Basel (EKBB, Switzerland), the National Institutional Review Board (NIMR, Tanzania), the Ifakara Health Institute IRB (IHIIRB, Tanzania), and the PATH’s Research Ethics Committee (REC, USA). Written informed consent was obtained from parents or guardians of participating children in accordance with the Declaration of Helsinki.

## Author Contributions

Substantial contributions to the conception or design of the work: GM, SR, CDa, CDo, and MJM. Acquisition of samples/data: GM, AN, MM, CJ, TR, JC, CDa, and CDo. Supervision analysis of samples: SR, KC. Analysis of data: GM, AA, JH, JA, DM, and CV. Data management: HS. Project management and coordination: ND-P and NW. Interpretation of data for the work and drafting the manuscript: GM, SR, CDo. Revising it critically for important intellectual content; final approval of the version to be published; and agreement to be accountable for all aspects of the work in ensuring that questions related to the accuracy or integrity of any part of the work are appropriately investigated and resolved: GM, SR, AA, AN, MM, KC, CJ, TR, JC, JH, HS, ND-P, NW, DM, JA, CV, CDa, CDo, MJM.

## Conflict of Interest Statement

The authors declare that the research was conducted in the absence of any commercial or financial relationships that could be construed as a potential conflict of interest.
