# Supplementary material for: RTS,S/AS01E Malaria Vaccine Induces Memory and Polyfunctional T Cell Responses in a Pediatric African Phase III Trial
Source: Front Immunol. 2017 Aug 23;8:1008. doi: 10.3389/fimmu.2017.01008 (PMC5572329; doi:10.3389/fimmu.2017.01008)
Supplement: Supplementary file 1 [file Presentation_1.PDF]

Supplementary Material

RTS,S/AS01E Malaria Vaccine Induces Memory and Polyfunctional T Cell Responses in a Pediatric African Phase III Trial

Gemma Moncunill[1,2,3,a*], Stephen C. De Rosa[2,4,a], Aintzane Ayestaran[1], Augusto J. Nhabomba[3], Maximillian Mpina[5], Kristen W. Cohen[2], Chenjerai Jairoce[3], Tobias Rutishauser[6,7], Joseph J. Campo[1,3], Jaroslaw Harezlak[8], Héctor Sanz[1], Núria Díez-Padrisa[1], Nana Aba Williams[1], Daryl Morris[2], John J. Aponte[1], Clarissa Valim[9,10], Claudia Daubenberger[6,7], Carlota Dobaño[1,3,b], M. Juliana McElrath[2, 11,b]

[a,b] Shared authorships

[1] ISGlobal, Barcelona Ctr. Int. Health Res. (CRESIB), Hospital Clínic - Universitat de Barcelona, Barcelona, Spain.

[2] Vaccine and Infectious Disease Division, Fred Hutchinson Cancer Research Center, Seattle, WA, USA.

[3] Centro de Investigação em Saúde de Manhiça (CISM), Vila de Manhiça, Maputo, Mozambique.

[4] Department of Laboratory Medicine, University of Washington, Seattle, WA, USA

[5] Ifakara Health Institute. Bagamoyo Research and Training Centre, Bagamoyo, Tanzania.

[6] Swiss Tropical and Public Health Institute, Basel, Switzerland.

[7] University of Basel, Basel, Switzerland.

[8] Department of Epidemiology and Biostatistics, School of Public Health-Bloomington, Indiana University, IN, USA.

[9] Department of Osteopathic Medical Specialties, Michigan State University, East Lansing, MI, USA.

[10] Department of Immunology and Infectious Diseases, Harvard T.H. Chen School of Public Health. Boston MA, USA.

[11] Department of Medicine, University of Washington, Seattle, WA, US.

*** Correspondence**

Gemma Moncunill, PhD.

gemma.moncunill@isglobal.org

1 Supplementary Material and Methods

Sample Collection

Blood was collected in 5 mL sodium citrate (BD Vacutainer® CPT™) tubes and transported to the laboratory on site within 2 h, where PBMC were isolated and frozen within three hours according to SOP. PBMC were isolated by density gradient centrifugation, and viable cells were counted with a Countess automated cell counter (Life Technologies). A minimum of 5 million PBMC were cryopreserved in heat-inactivated fetal bovine serum (FBS GIBCO, INVITROGEN, Cat #16000044) containing 10% dimethyl sulfoxide (DMSO, Sigma Chemical Co., Cat # D2650) in a final concentration of 5x 10^6 cells/mL, kept in a Mr Frosty at -80ºC for 24 h for temperature controlled-rate freezing, and then stored in liquid nitrogen. Samples were shipped with dry shipper to a centralized lab (Fred Hutchinson Cancer Research Center, Seattle, USA) where the samples were thawed to perform the ICS assay.

PBMC Stimulations

PBMC were thawed, washed and resuspended at 2×106 cells/mL in complete RPMI (RPMI 1640 with 25mM HEPES buffer and 2mM L-glutamine (Gibco BRL Life Technologies; Cat # 22400-089) supplemented with 10% heat inactivated FBS (Gemini Benchmark; Cat #100-106) and 1% penicillin-streptomycin (Gibco BRL Life Technologies; Cat #15140-122) and L-glutamine (Gibco BRL Life Technologies; Cat #25030-081). Cell counts and viability were determined by Guava Easycyte counter (Millipore), and cells were rested in conical tubes in 37ºC 5% CO2 incubator overnight. After resting cells were counted and resuspended with complete media at 2.5-5×106 cells/mL and 200 µL/well plated in a U-bottomed 96 well plate. PBMC were stimulated with CSP (31 peptides) and HBsAg peptide (54 peptides) pools (15-mers, overlapping by 11 amino acids, each peptide at a final concentration of 1 µg/mL, purity >70%; Biosynthesis, Germany) (Supplementary Table 1). Both peptide pools had been validated with malaria naïve adults and adults reporting not having been vaccinated with Hepatitis B vaccine, and no false positive responses were detected. Up to 1 million PBMC were used per stimulation and stimulation conditions were prioritized in the following order: DMSO, CSP peptide pool, HBsAg peptide pool and SEB (Sigma Chemical Co.; Cat #S4881). Stimulations included 1 µg/mL of the costimulatory antibodies, CD28 and CD49d (BD Biosciences; Cat #347690), 10 µg/mL of brefeldin A (Sigma Chemical Co.; Cat #B-7651) and monensin (BD; Cat #554724, concentration is proprietary and dilution is provided by vendor). One of the ICS panels used for staining, included the CD107a marker that required addition during the stimulations at the appropriate titer. Cultures were incubated 6 h at 37ºC, 5% CO2. Following stimulation, 20µL of 20mM EDTA (Fisher Chemicals, Cat #O2793-500) and plates were stored protected from light at 4-8°C overnight. Samples were stimulated and stained in batches of 24 samples, with both timepoints of each subject within the same batch and batches were balanced by site and vaccine group. Each batch included one control sample with known CMV responses that was stimulated with a CMV peptide pool (15-mer peptides overlapping by 11 covering pp65 of CMV) for QA/QC purposes.

Supplementary Table 1. CSP and HBsAg peptides

Peptide number	CSP	HBsAg
1	MMAPDPNANPNANPN	MENITSGFLGPLLVL
2	NANPNANPNANPNAN	TSGFLGPLLVLQAGF
3	DPNANPNANPNKNNQ	LGPLLVLQAGFFLLT
4	NPNANPNKNNQGNGQ	LVLQAGFFLLTRILT
5	NPNKNNQGNGQGHNM	AGFFLLTRILTIPQS
6	NNQGNGQGHNMPNDP	LLTRILTIPQSLDSW
7	NGQGHNMPNDPNRNV	ILTIPQSLDSWWTSL
8	HNMPNDPNRNVDENA	PQSLDSWWTSLNFLG
9	NDPNRNVDENANANS	DSWWTSLNFLGGSPV
10	RNVDENANANSAVKN	TSLNFLGGSPVCLGQ
11	ENANANSAVKNNNNE	FLGGSPVCLGQNSQS
12	ANSAVKNNNNEEPSD	SPVCLGQNSQSPTSN
13	VKNNNNEEPSDKHIK	LGQNSQSPTSNHSPT
14	NNEEPSDKHIKEYLN	SQSPTSNHSPTSCPP
15	PSDKHIKEYLNKIQN	TSNHSPTSCPPICPG
16	HIKEYLNKIQNSLST	SPTSCPPICPGYRWM
17	YLNKIQNSLSTEWSP	CPPICPGYRWMCLRR
18	IQNSLSTEWSPCSVT	CPGYRWMCLRRFIIF
19	LSTEWSPCSVTCGNG	RWMCLRRFIIFLFIL
20	WSPCSVTCGNGIQVR	LRRFIIFLFILLLCL
21	SVTCGNGIQVRIKPG	IIFLFILLLCLIFLL
22	GNGIQVRIKPGSANK	FILLLCLIFLLVLLD
23	QVRIKPGSANKPKDE	LCLIFLLVLLDYQGM
24	KPGSANKPKDELDYA	FLLVLLDYQGMLPVC
25	ANKPKDELDYANDIE	LLDYQGMLPVCPLIP
26	KDELDYANDIEKKIC	QGMLPVCPLIPGSTT
27	DYANDIEKKICKMEK	PVCPLIPGSTTTNTG
28	DIEKKICKMEKCSSV	LIPGSTTTNTGPCKT
29	KICKMEKCSSVFNVV	STTTNTGPCKTCTTP
30	MEKCSSVFNVVNSSI	NTGPCKTCTTPAQGN
31	KCSSVFNVVNSSIGL	CKTCTTPAQGNSMFP
32		TTPAQGNSMFPSCCC
33		QGNSMFPSCCCTKPT
34		MFPSCCCTKPTDGNC
35		CCCTKPTDGNCTCIP
36		KPTDGNCTCIPIPSS
37		GNCTCIPIPSSWAFA
38		CIPIPSSWAFAKYLW
39		PSSWAFAKYLWEWAS
40		AFAKYLWEWASVRFS

41		YLWEWASVRFSWLSL
42		WASVRFSWLSLLVPF
43		RFSWLSLLVPFVQWF
44		LSLLVPFVQWFVGLS
45		VPFVQWFVGLSPTVW
46		QWFVGLSPTVWLSAI
47		GLSPTVWLSAIWMMW
48		TVWLSAIWMMWYWGP
49		SAIWMMWYWGPSLYS
50		MMWYWGPSLYSIVSP
51		WGPSLYSIVSPFIPL
52		LYSIVSPFIPLLPIF
53		VSPFIPLLPIFFCLW
54		FIPLLPIFFCLWVYI

Intracellular Cytokine Staining
After stimulations PBMC were stained in the same 96-well plates at room temperature with one of two 16-color ICS panels. Cell staining was performed as described (1). Briefly, PBMC were stained for 20 min using the AViD viability dye (Molecular Probes/Invitrogen, Cat #L34957) followed by a surface staining for 20 min with antibodies against CXCR5, PD-1, CD45RA, CD56, CCR7, CD14 for panel 1 (Supplementary Table 2) and γδTCR, CD14, CD56 for panel 2 (Supplementary Table 3). After fixing and permeabilizing the cells using FACS Lyse (BD Biosciences; Cat #349202) and FACS Perm II (BD Biosciences; Cat #340973) for 10 min each, the cells were stained for 30 min with CD4, CD3, CD8, CD154, IFN-γ, TNF-α, IL-2, IL-4, Granzyme B (GzB), IL-21 for panel 1 (Supplementary Table S2) and CD4, CD3, CD8, CD154, IFN-γ, TNF-α, IL-2, IL-4, GzB, IL-13, IL-17, IL-10 for panel 2 (Supplementary Table 3). After washing, cells were resuspended in 1% paraformaldehyde. Data was acquired using a BD LSR II flow cytometer (BD Biosciences) directly from the plates using a high throughput sampler and configured with ultraviolet (355nm, 20 mW), violet (405 nm, 100 mW), blue (488nm, 100 mW), green (532 nm, 150 mW), and red (628 nm, 200 mW) lasers.

Supplementary Table 2. Antibodies used in Panel 1

Antibody	Manufacturer	Catalog Number	Markers shared with Panel 2
Surface staining			
CD14 BV510	BioLegend	301842	Lineage
CD56 BV650	BioLegend	318343	Lineage
CXCR5 PE-eFluor610	eBioscience	61-9185-42	T_{FH}
PD-1 PE-Cy7	eBioscience	25-2799-42	T_{FH}
CCR7 BV785	BioLegend	353229	Memory/Differentiation
CD45RA APC H7	BD Biosciences	560674	Memory/Differentiation

Intracellular staining			
CD4 BUV395	BD Biosciences	563550	Lineage
IFN-γ V450	Becton Dickinson	560371	Functional
CD3 BV570	BioLegend	300436	Lineage
CD154 BV605	BioLegend	310825	Functional
CD8 BV711	BD Biosciences	563677	Lineage
TNFα FITC	eBioscience	11-7349-82	Functional
IL-4 PerCP-Cy5.5	BioLegend	500822	Functional
IL-2 PE	BD Biosciences	559334	Functional
IL-21 APC	Miltenyi Biotec	130-096-882	Functional
Granzyme B Alx700	BD Biosciences	560213	Functional

Supplementary Table 3. Antibodies used in Panel 2

Antibody	Manufacturer	Catalog Number	Markers shared with Panel 1
Co-culture (added during stimulation)			
CD107a BV786 [a]	BD Biosciences	563869	Functional
Surface staining			
gdTCR PE-CF594	BD Biosciences	562511	Lineage
CD14 BV510	BioLegend	301842	Lineage
CD56 BV650	BioLegend	318343	Lineage
Intracellular staining			
CD4 BUV395	BD Biosciences	563550	Lineage
IFN-γ V450	Becton Dickinson	560371	Functional
CD3 BV570	BioLegend	300436	Lineage
CD154 BV605	BioLegend	310825	Functional
CD8 BV711	BD Biosciences	563677	Lineage
TNF-α FITC	eBioscience	11-7349-82	Functional
IL-10 PerCP-Cy5.5	BioLegend	501418	Functional
IL-2 PE	BD Biosciences	559334	Functional
IL-4 PE Cy7	BioLegend	500824	Functional
IL-13 APC	BioLegend	501907	Functional
Granzyme B Alx700	BD Biosciences	560213	Functional
IL-17 eF780	eBioscience	47-7179-42	Functional

[a] added during stimulation

Statistical Analysis

Multivariate linear mixed effect model

The model that was fitted for continuous data was a linear mixed effect model where levels of the stimulation were nested within each subject of the study. The model was chosen based on the study design and data generated. The model included a subject-specific random slope for timepoint, and a random intercept associated with each subject. Next, a

random effect associated with the intercept was included in the model for each level of stimulation. Random effects were assumed to be independent due to converge problems for some markers with sparse data.
The parametrization of the mixed effect model is given by:

$$y = X\beta + Z\gamma + \varepsilon$$

where y represents univariate data, β is an unknown vector of fixed effects with known model matrix X, γ is an unknown vector of random effects with known model matrix Z, and ε is an unknown random error vector. In our study, the fixed effects included are the stimulation, time of visit and vaccination status, as well as all interaction between the 3 factors. The fixed effect part of the model is given by:

$$\begin{aligned} log_{10}proportion &= \beta_0 + \beta_1 Stimulation + \beta_2 vaccination + \beta_3 time \\ &+ \beta_4 stimulation{:}time + \beta_5 vaccination{:}time + \beta_6 vaccination{:}time \\ &+ \beta_7 vaccination{:}stimulation{:}time \end{aligned}$$

where the $\log_{10}$ proportion is the logarithm 10 transformed proportion between the expressed cells and the total number of cells; variable Stimulation has two levels, the stimulation of interest and the background noise; vaccination has two levels too, RTS,S/AS01E and comparator vaccines and visit is a binary variable that corresponds to the baseline visit (pre-vaccination) or one month post-vaccination.
Linear mixed model were fitted by means of the function lme from package nlme. The nested random effects were defined by the command list(pid =pdDiag(~ timepoint), stimulation = ~1) and maximum likelihood approach was used to test the significance of fixed effects.
When the multivariate model was fitted, each one of the coefficients of the model has an interpretable meaning related to some questions of the interest of the study. The linear combination of coefficients that gives the answer to the scientific questions is obtained through the identification of the terms of the model that are involved.

- Stimulation specific effect at M3: $\beta_6 + \beta_7$
- Stimulation specific effect at M0: β_6
- Stimulation specific effect change from M0 to M3, RTSS-Comp: β_7
- Stimulation specific effect change from M0 to M3, RTSS: $\beta_4 + \beta_7$
- Stimulation specific effect change from M0 to M3, Comp: β_4
- Stimulation unspecific effect at M3: $\beta_2 + \beta_5 + \beta_6 + \beta_7$
- Background effect at M3: $\beta_2 + \beta_5$
- Stimulation unspecific effect at M0: $\beta_2 + \beta_6$
- Background effect at m0: β_2
- Stimulation unspecific effect change from M0 to M3, RTSS-Comp: $\beta_5 + \beta_7$
- Background effect change from M0 to M3, RTSS-Comp: β_5
- Stimulation unspecific effect change from M0 to M3, RTSS: $\beta_3 + \beta_4 + \beta_5 + \beta_7$
- Background effect change from M0 to M3, RTSS: $\beta_3 + \beta_5$
- Stimulation unspecific effect change from M0 to M3, Comp: $\beta_3 + \beta_4$
- Background effect change from M0 to M3, Comp: β_3
-

Coefficients, standard errors and confidence intervals for linear combinations of the hypothesis, were computed thought glht function from multicomp package. P values were

obtained though likelihood ratio test and percent change of the coefficients was obtained though the formula $100 * (10^{\beta} - 1)$.

Multiple Comparison Procedures
Adjustments for multiple testing were done through Holm (2) and Benjamini-Hochberg (3) approaches depending on the number of variables of the endpoints detailed in Table S4.

Supplementary Table 4. Description of endpoints and multiple testing approaches.

Endpoint	Description	Number of comparisons	Multiple testing method
1	Marginal cytokine responses and "IL2 OR TNF OR CD154" in $CD4^+$ T cells	7	Holm
2	Marginal cytokine responses and "IL2 OR TNF OR CD154" in $CD8^+$ T cells	7	Holm
3	Marginal cytokine responses in $CD4^+$ T cells extra markers Panel 1 and Panel 2	5	Holm
4	Marginal cytokine responses in $CD8^+$ T cells extra markers Panel 1 and Panel 2	5	Holm
5	Marginal cytokine responses in memory $CD4^+$ T cell subsets (N, CM, EM, TD)	7*4=28	Benjamini-Hochberg
6	Marginal cytokine responses in memory $CD8^+$ T cell subsets (N, CM, EM, TD)	7*4=28	Benjamini-Hochberg
7	Marginal cytokine responses in NK T cells, $CD4^-CD8^-$ cells and NK cells	7*3=21	Benjamini-Hochberg
8	Marginal cytokine responses in NK T cells, γδ T cells and NK cells	10*3=30	Benjamini-Hochberg

Abbreviations: N, naïve; CM, central memory; EM, effector memory; TD, terminally differentiated.

Statistical Software
All analyses were conducted using R software version 3.3.1 (2016-06-21) with the following functions: p.adjust function from stats package was used to compute Benjamini-Hochberg and Holm adjusted p values. For the positivity analysis, MIMOSA package (4) (version MIMOSA_1.10.2) was used; ConstructMIMOSAExpressionSet and MIMOSA functions were used to define the model, being the estimation method the Expectation-Maximization one. A two-sided Fisher exact test was performed to compare the positive responses from two vaccination groups based on fisher.test from stats package. For the continuous data analysis, the linear mixed effects models were fitted by means of the lme function from nlme package (5). Simultaneous inference of linear combinations of coefficients was done through the function glht from multcomp package (6). For the polyfunctional analysis, wilcox.test function from stats package was used to compare polyfunctionality scores between vaccination groups. Additional packages such as Hmisc, dplyr, tidyr, data.table and ReporteRs were used for data manipulation purposes (7-11). The VennDiagram package was used to generate the Venn plots.

References

1. Moncunill G, Dobaño C, McElrath MJ, De Rosa SC. OMIP-025: Evaluation of human T- and NK-cell responses including memory and follicular helper phenotype by intracellular cytokine staining. Cytom Part A (2015) 87:289–292. doi:10.1002/cyto.a.22590
2. Holm S. A Simple Sequentially Rejective Multiple Test Procedure. Scand J Stat (1979) 6:65–70. doi:10.2307/4615733
3. Benjamini Y, Hochberg Y. Benjamini Y, Hochberg Y. Controlling the false discovery rate: a practical and powerful approach to multiple testing. J R Stat Soc B (1995) 57:289–300. doi:10.2307/2346101
4. Finak G, Mcdavid A, Chattopadhyay P, Dominguez M, De Rosa S, Roederer M, Gottardo R. Mixture models for single-cell assays with applications to vaccine studies. Biostatistics (2014) 15:87–101. doi:10.1093/biostatistics/kxt024
5. Pinheiro J, Bates D, DebRoy S, Sarkar D and R Core Team (2016). _nlme: Linear and Nonlinear Mixed Effects Models. R package version 3.1-128, http://CRAN.R-project.org/package=nlme
6. Torsten Hothorn, Frank Bretz and Peter Westfall (2008). Simultaneous Inference in General Parametric Models. Biometrical Journal 50(3), 346--363
7. E Harrell Jr, with contributions from Charles Dupont and many others (2016). Hmisc: Harrell Miscellaneous. R package version 3.17-4. https://CRAN.R-project.org/package=Hmisc
8. Hadley Wickham and Romain Francois (2016). dplyr: A Grammar of Data Manipulation. R package version 0.5.0. https://CRAN.R-project.org/package=dplyr---
9. M Dowle, A Srinivasan, T Short, S Lianoglou with contributions from R Saporta and E Antonyan (2015). data.table: Extension of Data.frame. R package version 1.9.6. https://CRAN.R-project.org/package=data.table
10. Hadley Wickham (2016). tidyr: Easily Tidy Data with `spread()` and `gather()` functions. R package version 0.6.0. https://CRAN.R-project.org/package=tidyr
11. David Gohel (2016). ReporteRs: Microsoft Word, Microsoft PowerPoint and HTML Documents Generation. R package version 0.8.6. https://CRAN.R-project.org/package=ReporteRs
12. Hanbo Chen (2016). VennDiagram: Generate High-Resolution Venn and Euler Plots. R package version 1.6.17. https://CRAN.R-project.org/package=VennDiagram

2 Supplementary Results

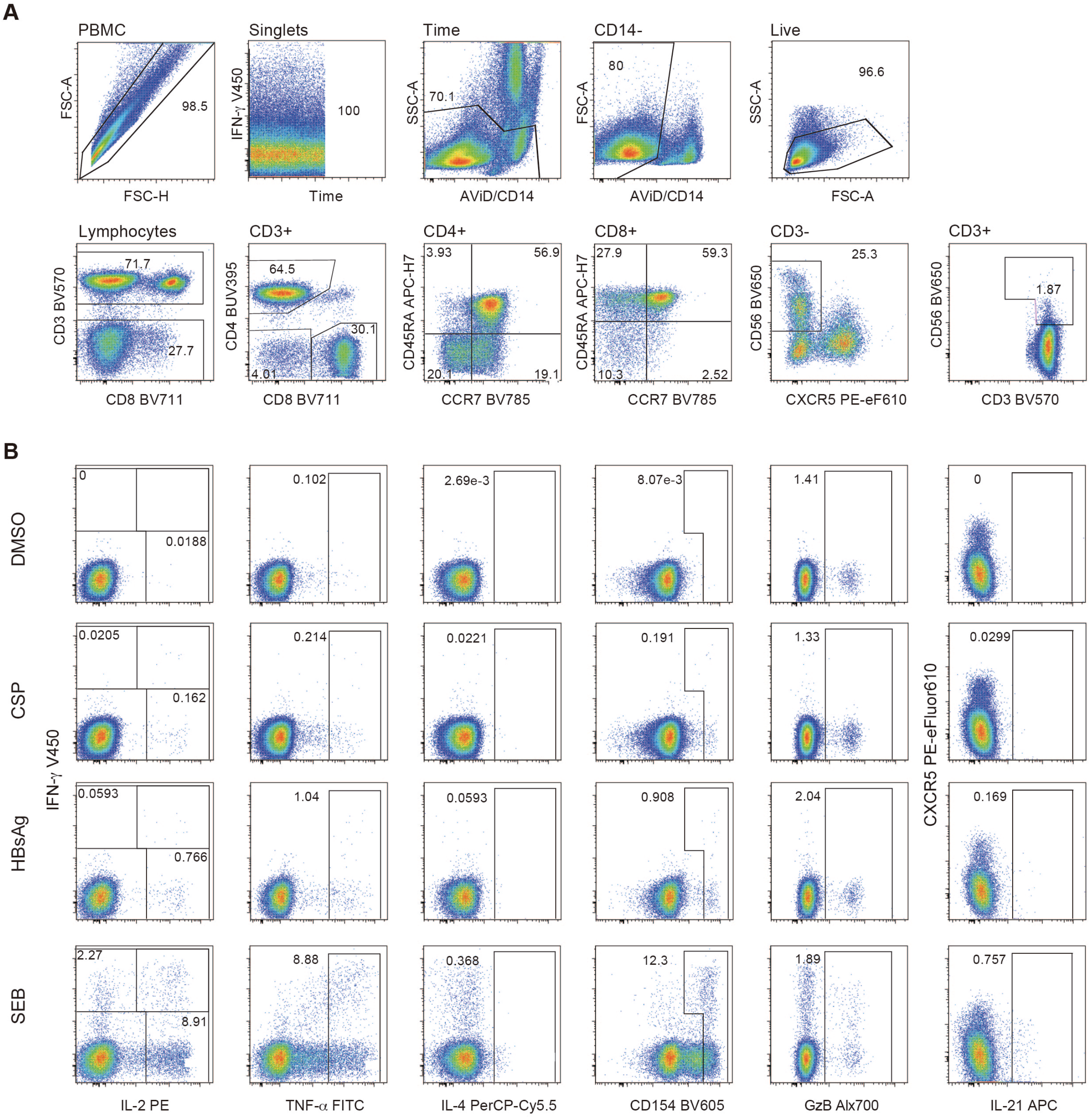

C

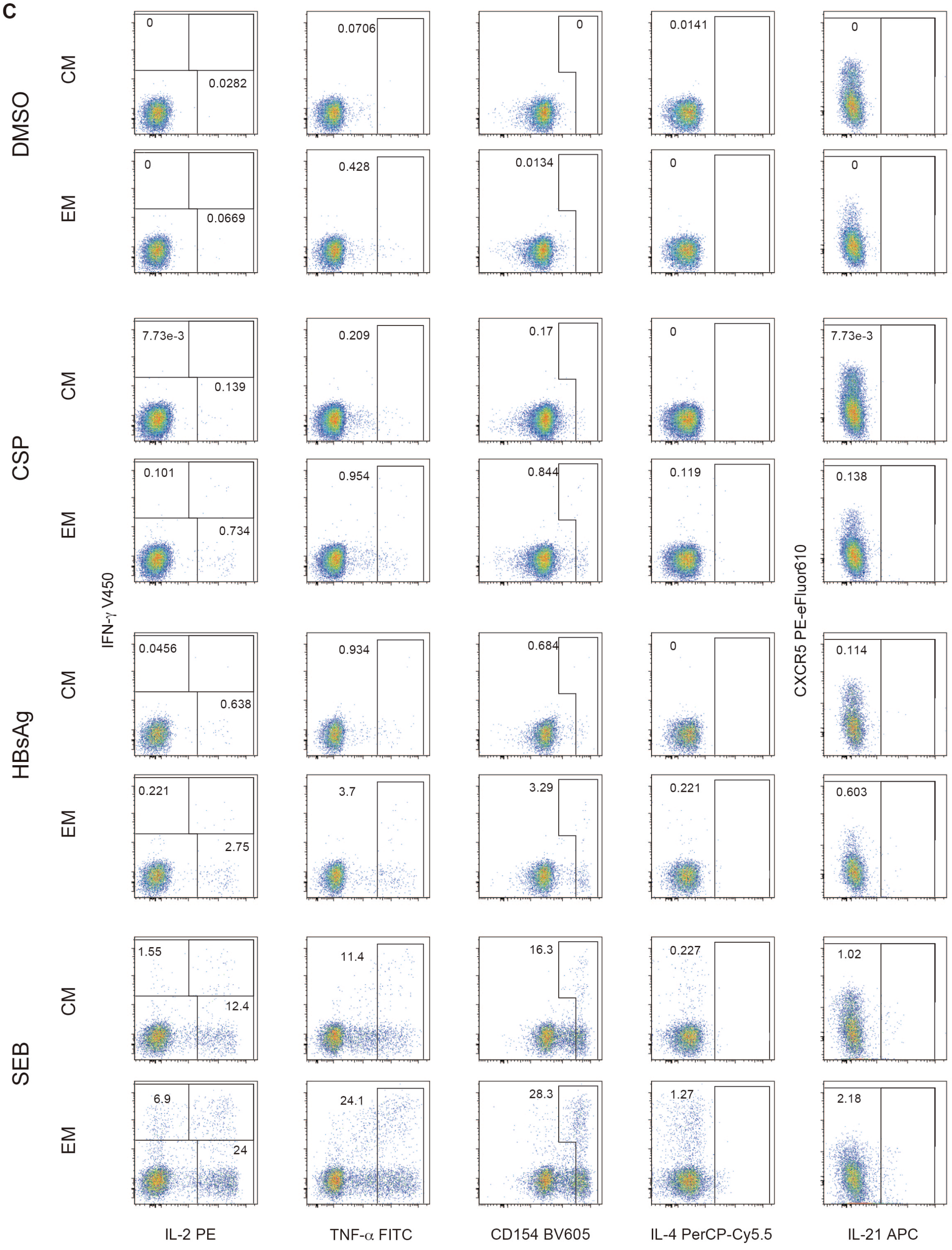
DMSO
CSP
HBsAg
SEB
CM
EM
IFN-γ V450
CXCR5 PE-eFluor610
IL-2 PE
TNF-α FITC
CD154 BV605
IL-4 PerCP-Cy5.5
IL-21 APC
0
0.0282
0.0706
0
0.0141
0
0
0.0669
0.428
0.0134
0
0
7.73e-3
0.139
0.209
0.17
0
7.73e-3
0.101
0.734
0.954
0.844
0.119
0.138
0.0456
0.638
0.934
0.684
0
0.114
0.221
2.75
3.7
3.29
0.221
0.603
1.55
12.4
11.4
16.3
0.227
1.02
6.9
24
24.1
28.3
1.27
2.18

Supplementary Figure 1. Gating strategy for panel 1 and example of RTS,S/AS01E vaccinated child with positive responses. All gates for nonfunctional markers were defined using fluorescence minus one (FMO) controls whereas gates for functional markers were defined using the unstimulated samples (after ensuring gates were above FMO thresholds). (A) Gating hierarchy to identify $CD4^{+}$ and $CD8^{+}$ T cells, $CD4^{-}CD8^{-}$ T cells, NK cells and NKT-like cells. Initial gating was done on FSC-H and FSC-A to discriminate singlets, followed by the exclusion of events collected during a period of time early in collection when fluctuations may occur. In this example, there were no problems of fluctuations and the time gate was minimized to avoid exclusion of any events. Monocytes and dead cells were excluded by the CD14 marker and an amine reactive dye in the same dump channel. Lymphocytes were gated using FSC-A and SSC-A. Within the gate of lymphocytes, $CD3^{+}$ cells were identified, followed by identification of $CD4^{+}$ and $CD8^{+}$ T cells. The expression level of CCR7 and CD45RA was examined within $CD4^{+}$ and $CD8^{+}$ T-cell subsets to later provide insight into the memory phenotype of the antigen specific cells. Identification of NK cells and NKT-like cells was performed using CD56 expression within $CD3^{-}$ and $CD3^{+}$ gates, respectively. (B) Functional marker expression on $CD4^{+}$ T cells after stimulation with DMSO (negative control), CSP peptides, HBsAg peptides, and SEB (positive control). A gate was applied for each cytokine, not taking into account the coexpression of other markers. Boolean gates were then created based on these gates to identify cells expressing different combinations of markers. (C) Functional markers for central memory (CM) and effector memory (EM) CD4+ T cells for the stimulations with DMSO (negative control), CSP peptides, HBsAg peptides and SEB (positive control).

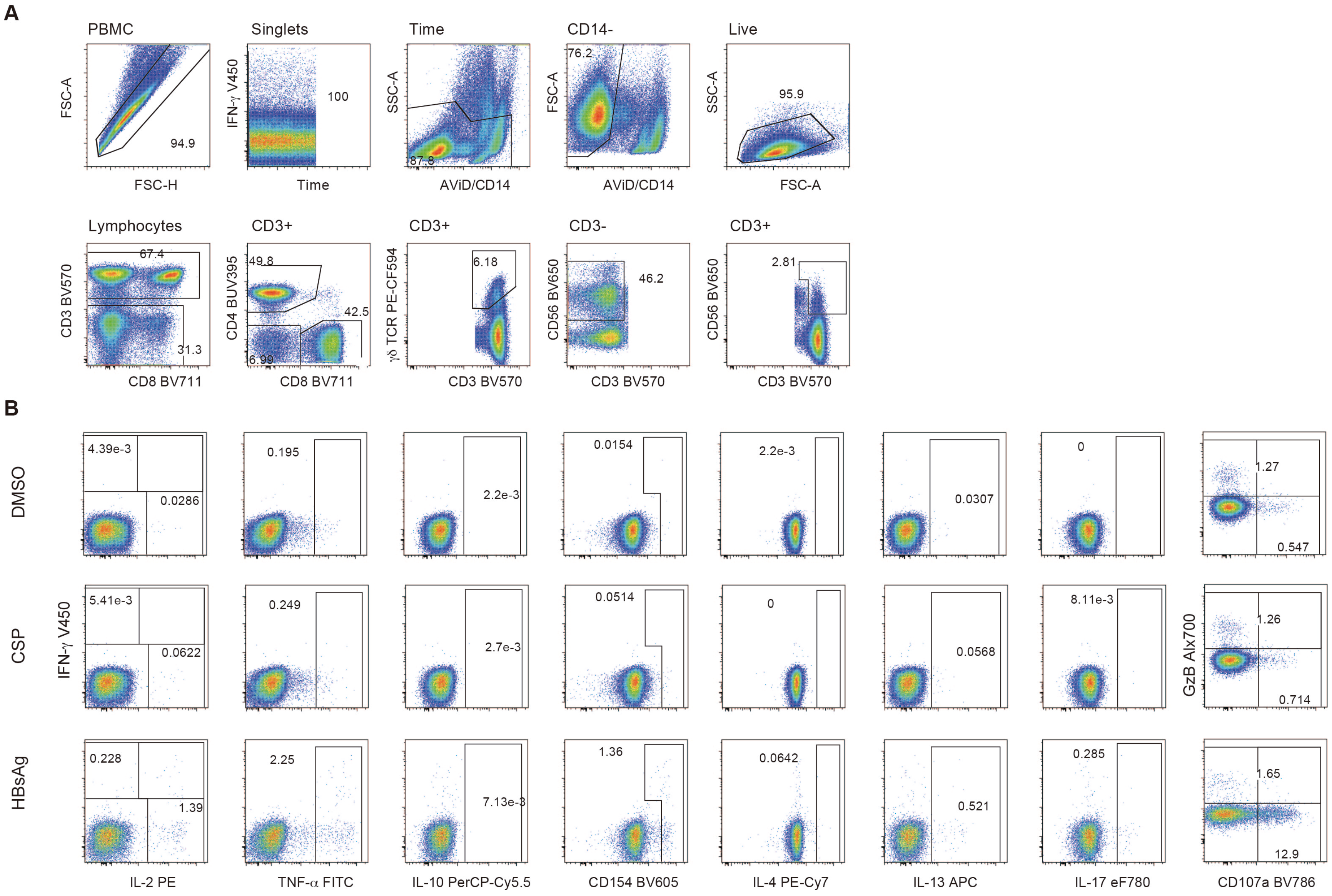
A
PBMC
FSC-A
FSC-H
94.9
Singlets
IFN-γ V450
Time
100
Time
SSC-A
AViD/CD14
87.8
CD14-
FSC-A
AViD/CD14
76.2
Live
SSC-A
FSC-A
95.9
Lymphocytes
CD3 BV570
CD8 BV711
67.4
31.3
CD3+
CD4 BUV395
CD8 BV711
49.8
42.5
6.99
CD3+
γδ TCR PE-CF594
CD3 BV570
6.18
CD3-
CD56 BV650
CD3 BV570
46.2
CD3+
CD56 BV650
CD3 BV570
2.81
B
DMSO
CSP
HBsAg
IFN-γ V450
GzB Alx700
4.39e-3
0.0286
0.195
2.2e-3
0.0154
2.2e-3
0.0307
0
1.27
0.547
5.41e-3
0.0622
0.249
2.7e-3
0.0514
0
0.0568
8.11e-3
1.26
0.714
0.228
1.39
2.25
7.13e-3
1.36
0.0642
0.521
0.285
1.65
12.9
IL-2 PE
TNF-α FITC
IL-10 PerCP-Cy5.5
CD154 BV605
IL-4 PE-Cy7
IL-13 APC
IL-17 eF780
CD107a BV786

Supplementary Figure 2. Gating strategy for panel 2 and example of RTS,S vaccinated child with positive responses. All gates for nonfunctional markers were defined using fluorescence minus one (FMO) controls whereas gates for functional markers were defined using the unstimulated samples (after ensuring gates were above FMO thresholds). (A) Gating hierarchy to identify $CD4^+$ and $CD8^+$ T cells, γδ-T cells, NK cells and NKT-like cells, Initial gating was done on FSC-H and FSC-A to discriminate singlets, followed by the exclusion of events collected during a period of time early in collection when fluctuations may occur. In this example, there were no problems of fluctuations and the time gate was minimized to avoid exclusion of any events. Monocytes and dead cells were excluded by the CD14 marker and an amine reactive dye in the same dump channel. Lymphocytes were gated using FSC-A and SSC-A. Within the gate of lymphocytes, $CD3^+$ cells were identified, followed by identification of $CD4^+$ and $CD8^+$ T cells and γδ-T cells. Identification of NK cells and NKT-like cells was performed using CD56 expression within $CD3^-$ and $CD3^+$ gates, respectively. (B) Functional markers for CD4+ T cells for the stimulations with DMSO (negative control), CSP peptides, and HBsAg peptides. A gate was applied for each cytokine, not taking into account the coexpression of other markers. Boolean gates were then created based on these gates to identify cells expressing different combinations of markers.

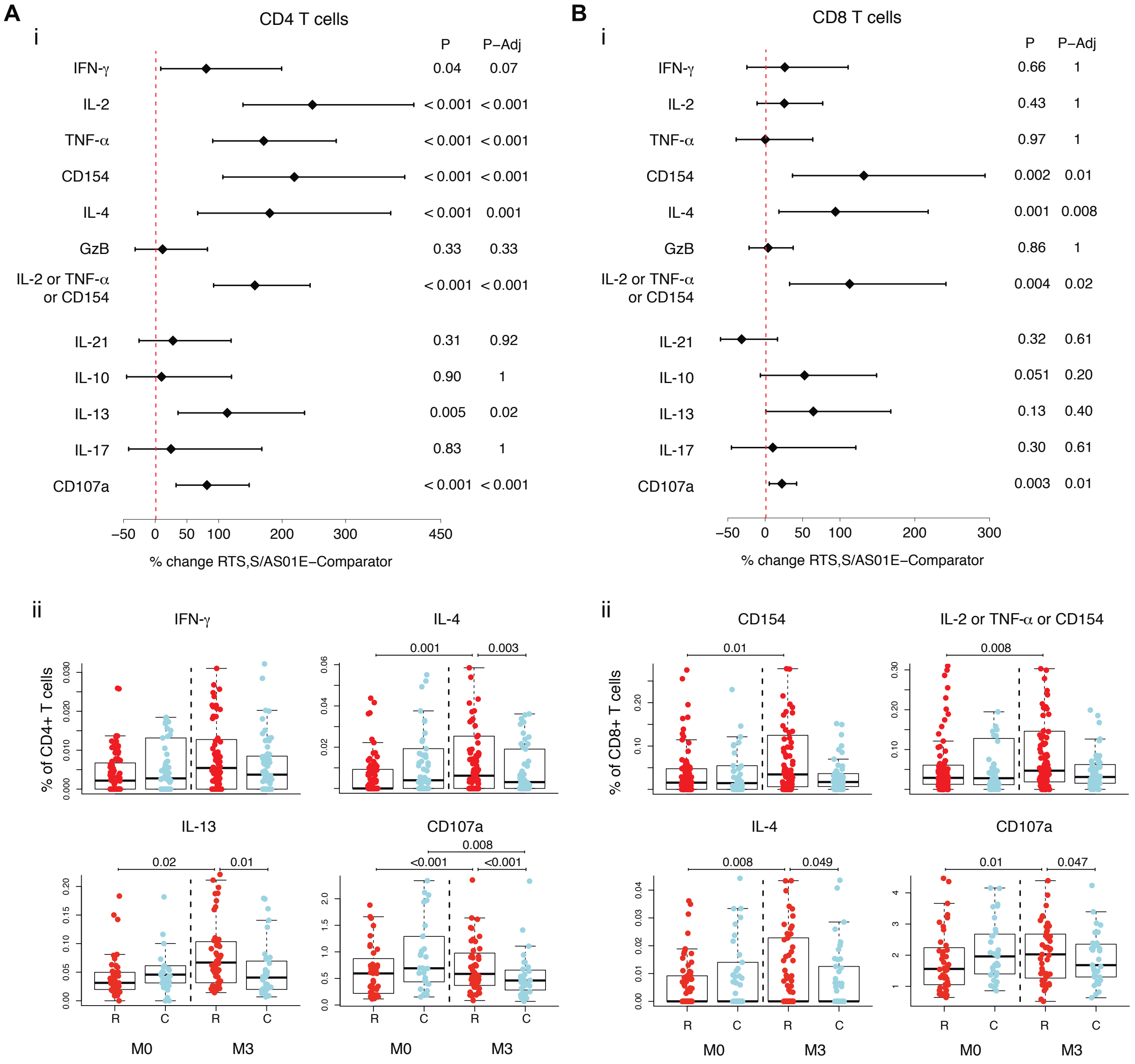

Supplementary Figure 3. CD4$^+$ and CD8$^+$ T cell responses following CSP stimulation when not taking background into account. CD4$^+$ (A) and CD8$^+$ (B) T cells expressing the common functional markers of both ICS panels. (i) Forest plot showing the effect of RTS,S/AS01E (R) vaccination from baseline (M0) to one month post-third immunization (M3), taking into account the M0 to M3 changes in comparator (C) vaccinees. The % change between RTS,S/AS01E and comparator vaccinees taking into account M0 to M3 changes and 95% confidence intervals shown were obtained with a multivariate linear mixed effect model. P-values (P) were obtained through likelihood ratio test and were adjusted for multiple testing (P-adj) through Holms approach. (ii) Box plots showing the frequencies of cells expressing the functional markers found to be statistically significant in i). Boxplots illustrate the medians and the 25th and 75th quartiles, and whiskers display 1.5 times interquartile ranges, outliers are not shown. Differences between vaccine groups at

M0 and at M3 and differences from M0 to M3 within each vaccine group were computed through a multivariate linear mixed effect model and P-values obtained through likelihood ratio test and were adjusted for multiple testing through Holms approach. Only significant P-values adjusted for multiple testing are shown. Sample size in (A) for markers detected by both staining panels N= 165 at M0 and 170 at M3. For comparisons including M0 and M3, only subjects that had samples at both timepoints are included (N=156 for markers detected by both panels, N=83 for markers detected by panel 1, N= 73 for markers detected by panel 2). Sample size in (B) N= 166 M0 and 169 at M3. For comparisons including M0 and M3, only subjects that had samples at both timepoints are included (N=157 for markers detected by both panels, N=84 for markers detected by panel 1, N= 73 for markers detected by panel 2).

Supplementary Table 5. Effect or RTS,S/AS01E vaccination on frequencies of all CSP-specific cells subsets taking into account frequencies at baseline and in comparator vaccinees.

Endpoint	ICS panel	Cell subset	Functional marker	% Change	SD	Lower CI	Upper CI	Raw P [a]	Adj P [b]
1	P1 & P2	$CD4^+$ T cell	IFN-γ	22.75	40.96	-37.37	140.58	0.55	0.55
			IL-2	216.98	27.21	97.78	408.02	< 0.001	< 0.001
			TNF-α	72.66	11.40	39.74	113.33	< 0.001	< 0.001
			CD154	101.44	31.29	18.15	243.45	0.01	0.043
			IL-4	35.97	32.06	-21.16	134.49	0.27	0.54
			GzB	17.73	11.61	-5.07	46.02	0.14	0.42
			IL-2 or TNF-α or CD154	53.89	9.58	28.63	84.12	< 0.001	< 0.001
2	P1 & P2	$CD8^+$ T cell	IFN-γ	18.30	31.57	-30.91	102.55	0.54	1
			IL-2	26.25	28.13	-22.33	105.24	0.35	1
			TNF-α	6.35	34.38	-40.41	89.80	0.83	1
			CD154	21.65	39.87	-36.98	134.84	0.40	1
			IL-4	-15.69	35.75	-53.68	53.47	0.58	1
			GzB	1.63	3.25	-4.54	8.20	0.61	1
			IL-2 or TNF-α or CD154	31.03	33.83	-25.99	131.96	0.35	1
3	P1	$CD4^+$ T cell	IL-21	79.63	46.95	-15.53	281.98	0.13	0.52
	P2		IL-10	23.63	58.01	-49.57	203.08	0.64	1
			IL-13	73.86	32.24	0.53	200.67	0.048	0.24
			IL-17	11.10	68.19	-59.90	207.81	0.84	1
			CD107a	-13.36	21.25	-40.61	26.38	0.46	1
4	P1	$CD8^+$ T cell	IL-21	-40.30	44.86	-71.13	23.45	0.17	0.83
	P2		IL-10	-16.36	29.93	-49.93	39.72	0.50	1
			IL-13	32.96	35.52	-26.72	141.24	0.35	1
			IL-17	-36.73	56.39	-73.66	52.01	0.31	1
			CD107a	-3.30	9.43	-18.96	15.39	0.71	1
5	P1	CM $CD4^+$ T cell	IFN-γ	-11.18	46.56	-58.01	87.89	0.76	0.88
			IL-2	119.30	44.64	6.38	352.09	0.03	0.16
			TNF-α	60.52	22.08	8.57	137.33	0.02	0.13
			CD154	88.94	47.86	-12.22	306.67	0.11	0.33
			IL-4	-23.46	36.75	-58.56	41.35	0.39	0.70
			IL-21	24.25	30.98	-26.78	110.86	0.42	0.70
			GzB	56.62	28.79	-4.62	157.17	0.08	0.28
		EM $CD4^+$ T cell	IFN-γ	1.52	49.67	-53.94	123.76	0.97	0.97
			IL-2	271.37	54.66	58.00	772.89	0.003	0.041
			TNF-α	75.28	19.69	23.24	149.30	0.003	0.041

Endpoint	ICS panel	Cell subset	Functional marker	% Change	SD	Lower CI	Upper CI	Raw P [a]	Adj P [b]
			CD154	221.02	59.80	28.10	704.52	0.01	0.13
			IL-4	115.89	41.19	9.80	324.49	0.03	0.15
			IL-21	18.03	22.86	-21.16	76.69	0.24	0.55
			GzB	21.68	21.56	-17.01	78.42	0.32	0.68
		Naive $CD4^+$ T cell	IFN-γ	-18.34	36.60	-55.68	50.48	0.52	0.75
			IL-2	2.31	50.90	-54.32	129.17	0.96	0.97
			TNF-α	25.08	49.90	-43.43	176.55	0.60	0.76
			CD154	-2.60	51.71	-56.97	120.47	0.95	0.97
			IL-4	80.48	34.09	1.56	220.74	0.046	0.18
			IL-21	25.01	44.93	-39.60	158.70	0.55	0.75
			GzB	-11.82	15.27	-33.25	16.49	0.38	0.70
		TD $CD4^+$ T cell	IFN-γ	-5.74	20.40	-34.49	35.63	0.75	0.88
			IL-2	10.20	52.79	-51.98	152.92	0.82	0.92
			TNF-α	-25.32	65.24	-72.09	99.84	0.56	0.75
			CD154	-36.06	38.61	-66.28	21.26	0.17	0.44
			IL-4	-32.81	32.57	-61.33	16.76	0.16	0.44
			IL-21	-10.54	19.31	-36.71	26.45	0.53	0.75
			GzB	-10.24	14.38	-31.02	16.81	0.42	0.70
6	P1	CM $CD8^+$ T cell	IFN-γ	32.57	26.98	-16.99	111.71	0.24	0.96
			IL-2	37.41	22.24	-7.31	103.70	0.12	0.96
			TNF-α	59.08	23.88	4.55	142.05	0.03	0.88
			CD154	40.52	44.33	-31.54	188.44	0.35	0.97
			IL-4	30.62	32.48	-24.73	126.68	0.34	0.97
			IL-21	2.55	10.71	-16.00	25.19	0.80	1
			GzB	-2.21	14.94	-25.56	28.46	0.76	1
		EM $CD8^+$ T cell	IFN-γ	10.60	39.96	-42.77	113.74	0.76	1
			IL-2	11.07	15.99	-16.95	48.55	0.48	0.97
			TNF-α	2.15	45.67	-51.14	113.52	0.95	1
			CD154	10.37	57.63	-54.77	169.29	0.83	1
			IL-4	8.99	48.11	-49.53	135.35	0.83	1
			IL-21	-11.31	17.65	-35.50	21.95	0.46	0.97
			GzB	-8.94	7.60	-21.13	5.12	0.20	0.96
		Naive $CD8^+$ T cell	IFN-γ	-11.53	29.29	-46.53	46.37	0.63	1
			IL-2	-1.99	30.07	-41.46	64.08	0.94	1
			TNF-α	0.06	21.40	-31.58	46.34	1	1
			CD154	20.87	55.49	-49.12	187.13	0.67	1
			IL-4	-37.06	46.26	-70.13	32.61	0.22	0.96
			IL-21	-41.07	53.41	-74.53	36.33	0.22	0.96

Endpoint	ICS panel	Cell subset	Functional marker	% Change	SD	Lower CI	Upper CI	Raw P [a]	Adj P [b]
			GzB	11.61	9.17	-6.02	32.56	0.21	0.96
		TD CD8$^+$ T cell	IFN-γ	0.89	39.85	-47.71	94.68	0.98	1
			IL-2	-13.54	17.05	-36.50	17.72	0.35	0.97
			TNF-α	20.87	50.64	-45.85	169.81	0.64	1
			CD154	-4.70	56.07	-60.17	128.02	0.91	1
			IL-4	-26.29	44.66	-64.25	52.00	0.41	0.97
			IL-21	0.26	40.62	-48.60	95.55	0.99	1
			GzB	3.69	5.30	-6.28	14.73	0.48	0.97
7	P1	NK cell	IFN-γ	-9.48	23.08	-39.74	35.98	0.63	0.79
			IL-2	51.38	25.78	-3.43	137.32	0.07	0.61
			TNF-α	1.98	42.40	-48.99	103.89	0.96	0.96
			CD154	27.19	14.32	-2.16	65.35	0.07	0.61
			IL-4	48.13	28.43	-9.29	141.90	0.12	0.61
			IL-21	17.03	29.28	-29.25	93.61	0.54	0.76
			GzB	10.32	9.63	-7.87	32.12	0.29	0.71
		CD4$^-$CD8$^-$T cell	IFN-γ	23.93	71.90	-57.14	258.34	0.64	0.79
			IL-2	-41.10	54.53	-74.90	38.22	0.23	0.71
			TNF-α	-17.83	65.00	-69.20	119.27	0.70	0.81
			CD154	-43.38	70.57	-80.12	61.25	0.30	0.71
			IL-4	42.93	73.39	-51.40	320.35	0.52	0.76
			IL-21	52.09	59.64	-39.20	280.42	0.37	0.71
			GzB	2.78	3.08	-3.16	9.08	0.37	0.71
		NKT cells	IFN-γ	111.11	71.29	-26.48	506.19	0.17	0.71
			IL-2	-12.91	66.85	-68.07	137.54	0.79	0.83
			TNF-α	135.13	70.64	-17.50	570.12	0.11	0.61
			CD154	18.93	89.63	-66.07	316.84	0.79	0.83
			IL-4	-46.07	81.70	-83.27	73.86	0.30	0.71
			IL-21	-30.81	74.43	-76.75	105.88	0.51	0.76
			GzB	-4.11	5.61	-13.84	6.72	0.44	0.76
8	P2	NK cell	IFN-γ	-10.40	30.25	-46.62	50.40	0.68	0.82
			IL-2	24.87	28.54	-23.66	104.25	0.38	0.67
			TNF-α	-48.29	43.05	-74.37	4.32	0.07	0.59
			CD154	31.00	18.70	-6.38	83.31	0.12	0.59
			IL4	-39.36	42.69	-69.79	21.73	0.16	0.60
			GzB	-1.23	1.07	-3.28	0.86	0.25	0.60
			IL10	37.44	31.19	-19.26	133.98	0.24	0.60
			IL13	-4.03	9.68	-19.93	15.03	0.66	0.82
			IL17	18.76	29.38	-28.32	96.75	0.50	0.69

Endpoint	ICS panel	Cell subset	Functional marker	% Change	SD	Lower CI	Upper CI	Raw P [a]	Adj P [b]
			CD107a	4.29	6.37	-7.59	17.70	0.50	0.69
		$\gamma\delta^+$ T cell	IFN-γ	-27.91	47.44	-66.32	54.30	0.40	0.67
			IL-2	-26.42	46.48	-65.18	55.47	0.42	0.67
			TNF-α	42.82	37.98	-24.01	168.41	0.27	0.60
			CD154	-18.71	84.55	-75.54	170.15	0.74	0.85
			IL4	-57.68	38.69	-77.71	-19.65	0.01	0.16
			GzB	-8.03	6.82	-19.19	4.67	0.21	0.60
			IL10	-52.90	61.70	-81.64	20.81	0.12	0.59
			IL13	-6.43	60.75	-63.09	137.23	0.89	0.92
			IL17	4.73	41.05	-46.62	105.51	0.89	0.92
			CD107a	34.73	28.40	-17.45	119.90	0.24	0.60
		NKT cell	IFN-γ	59.84	55.34	-32.58	278.99	0.29	0.60
			IL-2	-4.21	38.05	-49.08	80.20	0.89	0.92
			TNF-α	117.89	82.00	-32.62	604.62	0.19	0.60
			CD154	65.46	96.67	-56.05	522.87	0.46	0.69
			IL4	71.49	39.81	-11.08	230.74	0.11	0.59
			GzB	0.31	3.70	-6.59	7.71	0.93	0.93
			IL10	-21.49	80.67	-75.37	150.27	0.68	0.82
			IL13	-66.31	50.94	-84.96	-24.49	0.01	0.16
			IL17	21.32	20.33	-15.59	74.38	0.30	0.60
			CD107a	-15.83	22.69	-43.63	25.67	0.40	0.67

Abbreviations: SD, standard deviation; CI, confidence interval; CM, central memory; EM, effector memory; TD, terminally differentiated.

[a] P-values were computed through likelihood ratio test.

[b] P-values adjusted for multiple testing through Holm or Benjamini-Hochberg tests, depending on the endpoint (see Supplementary Table 4).

Supplementary Table 6. Effect or RTS,S/AS01E vaccination on frequencies of all HBsAg-specific cells subsets taking into account frequencies at baseline and in comparator vaccinees.

Endpoint	ICS panel	Cell subset	Functional marker	% Change	SD	Lower CI	Upper CI	Raw P [a]	Adj P [b]
1	P1 & P2	$CD4^+$ T cell	IFN-γ	52.03	46.55	-28.12	221.56	0.27	0.82

Endpoint	ICS panel	Cell subset	Functional marker	% Change	SD	Lower CI	Upper CI	Raw P [a]	Adj P [b]
			IL-2	793.18	41.86	350.11	1672.43	< 0.001	< 0.001
			TNF-α	346.40	28.87	171.54	633.87	< 0.001	< 0.001
			CD154	268.32	44.04	80.13	653.10	< 0.001	0.002
			IL-4	33.22	50.42	-40.16	196.55	0.48	0.93
			GzB	10.21	14.24	-15.10	43.08	0.47	0.93
			IL-2 or TNF-α or CD154	208.34	25.10	98.81	378.22	< 0.001	< 0.001
2	P1 & P2	$CD8^+$ T cell	IFN-γ	-37.09	49.01	-71.21	37.47	0.25	1
			IL-2	52.15	37.32	-18.29	183.30	0.19	1
			TNF-α	46.85	40.38	-24.46	185.48	0.26	1
			CD154	1.79	53.25	-55.91	135.01	0.97	1
			IL-4	-61.21	51.99	-82.92	-11.88	0.02	0.17
			GzB	1.82	6.37	-9.79	14.92	0.77	1
			IL-2 or TNF-α or CD154	0.38	47.60	-53.20	115.29	0.99	1
3	P1	$CD4^+$ T cell	IL-21	9.54	65.39	-59.14	193.66	0.86	0.86
	P2		IL-10	274.56	81.60	16.32	1106.11	0.03	0.09
			IL-13	160.81	52.27	14.39	494.60	0.02	0.09
			IL-17	158.49	66.81	-5.17	604.65	0.07	0.13
4	P1	$CD8^+$ T cell	IL-21	-33.08	63.63	-74.51	75.67	0.42	0.78
	P2		IL-10	-37.59	41.07	-68.21	22.51	0.17	0.52
			IL-13	41.09	49.41	-35.78	209.96	0.39	0.78
			IL-17	-64.28	61.86	-86.10	-8.21	0.04	0.14
5	P1	CM $CD4^+$ T cell	IFN-γ	1.56	39.42	-47.05	94.81	0.96	0.96
			IL-2	468.02	83.20	73.39	1760.80	0.004	0.02
			TNF-α	184.57	47.03	33.68	505.76	0.007	0.03
			CD154	644.59	68.00	169.34	1958.41	< 0.001	0.002
			IL-4	-10.54	48.25	-58.65	93.53	0.78	0.90
			IL-21	45.24	44.39	-29.30	198.38	0.31	0.54
			GzB	116.44	89.89	-38.41	660.65	0.23	0.43
		EM $CD4^+$ T cell	IFN-γ	17.03	61.07	-54.02	197.88	0.74	0.90
			IL-2	621.53	70.83	152.59	1961.08	< 0.001	0.003
			TNF-α	342.81	58.93	78.60	997.90	0.002	0.009
			CD154	314.45	67.81	50.26	1043.18	0.007	0.03
			IL-4	96.18	45.04	-5.34	306.61	0.07	0.20
			IL-21	182.23	37.60	50.97	427.58	0.001	0.009
			GzB	59.91	36.11	-12.61	192.60	0.13	0.33
		Naive $CD4^+$ T cell	IFN-γ	-40.78	42.02	-70.22	17.79	0.14	0.33
			IL-2	232.50	69.12	18.72	831.23	0.03	0.08
			TNF-α	99.10	72.53	-31.64	479.84	0.21	0.43

Endpoint	ICS panel	Cell subset	Functional marker	% Change	SD	Lower CI	Upper CI	Raw P [a]	Adj P [b]
			CD154	-12.41	56.81	-63.73	111.51	0.77	0.90
			IL-4	-15.38	36.00	-53.68	54.58	0.59	0.82
			IL-21	-5.71	62.12	-63.42	143.06	0.91	0.94
			GzB	-27.99	29.36	-56.52	19.27	0.22	0.43
		TD $CD4^+$ T cell	IFN-γ	-11.57	10.13	-26.81	6.85	< 0.001	< 0.001
			IL-2	35.80	54.51	-42.12	218.61	0.48	0.71
			TNF-α	-10.62	78.04	-71.14	176.84	0.85	0.91
			CD154	-22.14	42.79	-61.26	56.51	0.48	0.71
			IL-4	-10.69	35.35	-50.66	61.65	0.71	0.90
			IL-21	-6.35	30.73	-44.61	58.34	0.81	0.90
			GzB	40.90	55.33	-40.57	234.03	0.44	0.71
6	P1	CM $CD8^+$ T cell	IFN-γ	17.39	28.81	-28.53	92.81	0.55	0.94
			IL-2	21.92	28.93	-25.91	100.61	0.44	0.87
			TNF-α	56.32	30.87	-7.75	164.87	0.1	0.69
			CD154	54.63	51.15	-31.19	247.50	0.29	0.82
			IL-4	4.44	24.17	-31.68	59.65	0.84	0.94
			IL-21	-4.51	16.89	-29.67	29.66	0.77	0.94
			GzB	9.83	24.78	-28.83	69.48	0.67	0.94
		EM $CD8^+$ T cell	IFN-γ	93.38	55.93	-19.03	361.88	0.14	0.71
			IL-2	-1.65	18.30	-29.25	36.71	0.92	0.94
			TNF-α	-3.24	53.00	-57.96	122.69	0.94	0.94
			CD154	-36.12	57.87	-73.89	56.32	0.33	0.82
			IL-4	-40.80	51.39	-73.74	33.45	0.21	0.71
			IL-21	-15.10	20.50	-41.09	22.36	0.38	0.82
			GzB	1.91	4.76	-6.97	11.64	0.69	0.94
		Naive $CD8^+$ T cell	IFN-γ	-4.69	48.66	-56.18	107.31	0.90	0.94
			IL-2	2.64	24.24	-32.92	57.06	0.90	0.94
			TNF-α	16.81	29.88	-30.02	94.98	0.55	0.94
			CD154	7.75	70.07	-61.95	205.10	0.89	0.94
			IL-4	-58.50	58.79	-83.24	2.72	0.058	0.60
			IL-21	-12.25	80.10	-72.30	178.02	0.82	0.94
			GzB	31.47	25.10	-15.25	103.92	0.23	0.71
		TD $CD8^+$ T cell	IFN-γ	47.41	54.41	-37.09	245.41	0.37	0.82
			IL-2	-26.88	25.98	-53.50	14.99	0.18	0.71
			TNF-α	108.16	48.38	-3.95	351.13	0.06	0.60
			CD154	-7.67	68.89	-66.94	157.88	0.88	0.94
			IL-4	-46.53	61.02	-78.98	36.01	0.19	0.71
			IL-21	-13.78	36.68	-53.27	59.09	0.64	0.94

Endpoint	ICS panel	Cell subset	Functional marker	% Change	SD	Lower CI	Upper CI	Raw P [a]	Adj P [b]
			GzB	7.22	3.78	-0.30	15.29	0.06	0.60
7	P1	NK cell	IFN-γ	-6.39	36.83	-49.37	73.07	0.83	0.88
			IL-2	47.84	24.04	-3.08	125.53	0.07	0.25
			TNF-α	-6.47	55.04	-60.40	120.91	0.88	0.88
			CD154	31.13	20.99	-9.73	90.50	0.16	0.47
			IL-4	59.30	21.00	9.64	131.44	0.02	0.11
			IL-21	54.69	47.50	-27.79	231.36	0.26	0.60
			GzB	0.57	1.67	-2.64	3.88	0.73	0.86
		$CD4^-CD8^-$T cell	IFN-γ	-33.92	89.22	-81.07	130.63	0.52	0.72
			IL-2	-37.81	71.71	-78.45	79.45	0.38	0.67
			TNF-α	61.09	82.86	-50.65	425.80	0.44	0.67
			CD154	-41.42	102.03	-85.24	132.49	0.45	0.67
			IL-4	-69.34	86.24	-90.94	3.74	0.059	0.25
			IL-21	66.42	80.33	-47.60	428.56	0.39	0.67
			GzB	1.14	8.04	-13.08	17.70	0.88	0.88
		NKT cells	IFN-γ	160.36	128.76	-48.57	1218.05	0.25	0.60
			IL-2	281.97	87.20	11.76	1205.46	0.04	0.19
			TNF-α	546.17	106.53	55.96	2577.27	0.01	0.11
			CD154	444.43	91.73	52.01	1849.86	0.01	0.11
			IL-4	-29.03	95.51	-80.93	164.09	0.61	0.79
			IL-21	-46.25	78.33	-82.70	67.01	0.29	0.60
			GzB	9.20	16.17	-18.61	46.49	0.64	0.79
8	P2	NK cell	IFN-γ	-2.54	50.14	-56.05	116.15	0.85	0.88
			IL-2	-19.49	32.96	-53.93	40.71	0.45	0.81
			TNF-α	-39.36	67.15	-77.84	65.96	0.33	0.81
			CD154	-23.43	28.22	-52.96	24.65	0.28	0.81
			IL-4	-15.86	81.54	-73.85	170.77	0.77	0.87
			GzB	-0.19	2.95	-5.72	5.65	0.94	0.94
			IL-10	16.16	53.05	-49.56	167.51	0.72	0.85
			IL-13	-6.27	14.59	-28.23	22.40	0.63	0.82
			IL-17	28.32	39.05	-32.75	144.85	0.45	0.81
		$\gamma\delta^+$ T cell	IFN-γ	-58.47	76.14	-86.31	25.95	0.12	0.81
			IL-2	64.81	55.51	-30.64	291.57	0.26	0.81
			TNF-α	37.38	51.45	-39.10	209.92	0.45	0.81
			CD154	-30.26	103.34	-82.65	180.27	0.61	0.82
			IL-4	-32.33	65.56	-74.81	81.79	0.44	0.81
			GzB	-1.95	9.94	-18.58	18.07	0.84	0.88
			IL-10	-71.12	71.39	-89.95	-16.97	0.02	0.62

Endpoint	ICS panel	Cell subset	Functional marker	% Change	SD	Lower CI	Upper CI	Raw P [a]	Adj P [b]
			IL-13	-34.90	84.48	-80.40	116.18	0.48	0.82
			IL-17	-16.43	56.16	-65.11	100.19	0.69	0.84
		NKT cells	IFN-γ	78.14	91.51	-50.15	536.58	0.38	0.81
			IL-2	89.11	69.09	-32.45	429.40	0.23	0.81
			TNF-α	94.92	105.31	-52.41	698.30	0.35	0.81
			CD154	96.35	109.11	-53.75	733.61	0.36	0.81
			IL-4	35.30	82.99	-58.61	342.26	0.62	0.82
			GzB	-20.41	12.98	-37.34	1.10	0.09	0.81
			IL-10	103.66	117.45	-55.57	833.49	0.36	0.81
			IL-13	-30.79	106.59	-83.31	186.95	0.62	0.82
			IL-17	-15.29	32.13	-50.93	46.25	0.56	0.82

Abbreviations: SD, standard deviation; CI, confidence interval; CM, central memory; EM, effector memory; TD, terminally differentiated.

[a] P-values were computed through likelihood ratio test.

[b] P-values adjusted for multiple testing through Holm or Benjamini-Hochberg tests, depending on the endpoint (see Supplementary Table 4).

Supplementary Table 7. Proportion of CSP and HBsAg positive responses in $CD4^+$ T cells at baseline

		CSP				HBsAg			
		RTS,S/AS01E	Comparator	P value		RTS,S/AS01E	Comparator	P value	
Functional marker	ICS panel	Positive/total (%)	Positive/total (%)	Raw P	Adj P	Positive/total (%)	Positive/total (%)	Raw P	Adj P
IFN-γ	P1 & P2	0/65 (0%)	0/100 (0%)	1	1	7/62 (11.29%)	0/36 (0%)	0.045	0.27
IL-2	P1 & P2	0/65 (0%)	0/100 (0%)	1	1	0/62 (0%)	4/36 (11.11%)	0.02	0.11
TNF-α	P1 & P2	0/65 (0%)	0/100 (0%)	1	1	0/62 (0%)	1/36 (2.78%)	0.37	1
CD154	P1 & P2	7/65 (10.77%)	7/100 (7%)	0.41	1	8/62 (12.9%)	4/36 (11.11%)	1	1
IL-4	P1 & P2	1/65 (1.54%)	1/100 (1%)	1	1	5/62 (8.06%)	4/36 (11.11%)	0.72	1
GzB	P1 & P2	6/65 (9.23%)	14/100 (14%)	0.47	1	22/62 (35.48%)	18/36 (50%)	0.2	1
IL-2 or TNF-α or CD154	P1 & P2	1/65 (1.54%)	1/100 (1%)	1	1	1/62 (1.61%)	2/36 (5.56%)	0.55	1
IL-21	P1	1/53 (1.89%)	0/35 (0%)	1	1	9/32 (28.12%)	4/18 (22.22%)	0.75	1
IL-10	P2	0/47 (0%)	0/30 (0%)	1	1	1/30 (3.33%)	2/18 (11.11%)	0.55	1
IL-13	P2	0/47 (0%)	0/30 (0%)	1	1	7/30 (23.33%)	8/18 (44.44%)	0.2	0.79
IL-17	P2	0/47 (0%)	0/30 (0%)	1	1	0/30 (0%)	0/18 (0%)	1	1

[a] Raw P, original P-value computed based on two-sided Fisher's exact test.
[b] Adj P, P-values adjusted for multiple testing through Holm approach.

Supplementary Table 8. Proportion of CSP and HBsAg positive responses in memory CD4$^+$ T cell subsets at baseline

		CSP				HBsAg			
		RTS,S/AS01E	Comparator	P value		RTS,S/AS01E	Comparator	P value	
Memory subset	Functional marker	Positive/total (%)	Positive/total (%)	Raw P [a]	Adj P [b]	Positive/total (%)	Positive/total (%)	Raw P [a]	Adj P [b]
CM	IFN-γ	0/53 (0%)	0/35 (0%)	1	1	0/32 (0%)	0/18 (0%)	1	1
	IL-2	0/53 (0%)	0/35 (0%)	1	1	0/32 (0%)	0/18 (0%)	1	1
	TNF-α	0/53 (0%)	0/35 (0%)	1	1	0/32 (0%)	0/18 (0%)	1	1
	CD154	0/53 (0%)	0/35 (0%)	1	1	0/32 (0%)	0/18 (0%)	1	1
	IL-4	0/53 (0%)	0/35 (0%)	1	1	0/32 (0%)	0/18 (0%)	1	1
	IL-21	0/53 (0%)	0/35 (0%)	1	1	0/32 (0%)	0/18 (0%)	1	1
	GzB	3/53 (5.66%)	3/35 (8.57%)	0.68	1	4/32 (12.5%)	3/18 (16.67%)	0.69	1
EM	IFN-γ	0/53 (0%)	0/35 (0%)	1	1	0/32 (0%)	0/18 (0%)	1	1
	IL-2	0/53 (0%)	0/35 (0%)	1	1	0/32 (0%)	1/18 (5.56%)	0.36	1
	TNF-α	0/53 (0%)	0/35 (0%)	1	1	0/32 (0%)	0/18 (0%)	1	1
	CD154	0/53 (0%)	0/35 (0%)	1	1	0/32 (0%)	0/18 (0%)	1	1
	IL-4	0/53 (0%)	0/35 (0%)	1	1	0/32 (0%)	0/18 (0%)	1	1
	IL-21	0/53 (0%)	0/35 (0%)	1	1	0/32 (0%)	0/18 (0%)	1	1
	GzB	0/53 (0%)	1/35 (2.86%)	0.4	1	1/32 (3.12%)	0/18 (0%)	1	1
TD	IFN-γ	0/53 (0%)	0/35 (0%)	1	1	2/32 (6.25%)	0/18 (0%)	0.53	1
	IL-2	0/53 (0%)	0/35 (0%)	1	1	0/32 (0%)	0/18 (0%)	1	1
	TNF-α	0/53 (0%)	0/35 (0%)	1	1	0/32 (0%)	0/18 (0%)	1	1
	CD154	2/53 (3.77%)	2/35 (5.71%)	1	1	2/32 (6.25%)	1/18 (5.56%)	1	1
	IL-4	0/53 (0%)	0/35 (0%)	1	1	0/32 (0%)	0/18 (0%)	1	1
	IL-21	1/53 (1.89%)	0/35 (0%)	1	1	9/32 (28.12%)	4/18 (22.22%)	0.75	1
	GzB	9/53 (16.98%)	5/35 (14.29%)	1	1	7/32 (21.88%)	2/18 (11.11%)	0.46	1
Naive	IFN-γ	0/53 (0%)	0/35 (0%)	1	1	0/32 (0%)	0/18 (0%)	1	1

	IL-2	0/53 (0%)	0/35 (0%)	1	1	0/32 (0%)	0/18 (0%)	1	1
	TNF-α	0/53 (0%)	0/35 (0%)	1	1	0/32 (0%)	0/18 (0%)	1	1
	CD154	0/53 (0%)	0/35 (0%)	1	1	0/32 (0%)	0/18 (0%)	1	1
	IL-4	0/53 (0%)	0/35 (0%)	1	1	0/32 (0%)	0/18 (0%)	1	1
	IL-21	0/53 (0%)	0/35 (0%)	1	1	3/32 (9.38%)	3/18 (16.67%)	0.65	1
	GzB	0/53 (0%)	0/35 (0%)	1	1	0/32 (0%)	0/18 (0%)	1	1

Abbreviations: CM, central memory; EM, effector memory; TD, terminally differentiated
[a] Raw P, original P-value computed based on two-sided Fisher's exact test.
[b] Adj P, P-values adjusted for multiple testing through Benjamini-Hochberg approach.

Supplementary Table 9. Proportion of CD8^{+}T cell positive responses for CSP and HBsAg at post-vaccination.

		**CSP**				**HBsAg**			
		**Comparator**	**RTS,S/AS01E**	**P value**		**Comparator**	**RTS,S/AS01E**	**P value**	
**Functional marker**	**ICS panel**	**Positive/total (%)**	**Positive/total (%)**	**Raw P** [a]	**Adj P** [b]	**Positive/total (%)**	**Positive/total (%)**	**Raw P** [a]	**Adj P** [b]
IFN-γ	P1 & P2	0/69 (0%)	0/99 (0%)	1	1	1/50 (2%)	4/67 (5.97%)	0.39	1
IL-2	P1 & P2	0/69 (0%)	0/99 (0%)	1	1	0/50 (0%)	1/67 (1.49%)	1	1
TNF-α	P1 & P2	0/69 (0%)	0/99 (0%)	1	1	0/50 (0%)	0/67 (0%)	1	1
CD154	P1 & P2	3/69 (4.35%)	9/99 (9.09%)	0.36	1	10/50 (20%)	11/67 (16.42%)	0.63	1
IL-4	P1 & P2	1/69 (1.45%)	0/99 (0%)	0.41	1	4/50 (8%)	3/67 (4.48%)	0.46	1
GzB	P1 & P2	12/69 (17.39%)	14/99 (14.14%)	0.67	1	50/50 (100%)	67/67 (100%)	1	1
IL-2 or TNF-α or CD154	P1 & P2	1/69 (1.45%)	3/99 (3.03%)	0.64	1	7/50 (14%)	7/67 (10.45%)	0.58	1
IL-21	P1	0/38 (0%)	0/52 (0%)	1	1	6/27 (22.22%)	6/37 (16.22%)	0.75	1

IL-10	P2	0/31 (0%)	1/47 (2.13%)	1	1	5/23 (21.74%)	9/30 (30%)	0.55	1
IL-13	P2	0/31 (0%)	2/47 (4.26%)	0.51	1	6/23 (26.09%)	16/30 (53.33%)	0.055	0.22
IL-17	P2	0/31 (0%)	1/47 (2.13%)	1	1	3/23 (13.04%)	4/30 (13.33%)	1	1

[a] Raw P, original P-value computed based on two-sided Fisher's exact test.
[b] Adj P, P-values adjusted for multiple testing through Holm approach.

Supplementary Table 10. Proportion of memory CD8$^+$ T cell positive responses for CSP and HBsAg at post-vaccination.

		**CSP**				**HBsAg**			
		**Comparator**	**RTS,S/AS01E**	**P value**		**Comparator**	**RTS,S/AS01E**	**P value**	
**Memory subset**	**Functional marker**	**Positive/total (%)**	**Positive/total (%)**	**Raw P** [a]	**Adj P** [b]	**Positive/total (%)**	**Positive/total (%)**	**Raw P** [a]	**Adj P** [b]
CM	IFN-γ	0/38 (0%)	0/52 (0%)	1	1	0/27 (0%)	0/37 (0%)	1	1
	IL-2	0/38 (0%)	0/52 (0%)	1	1	0/27 (0%)	0/37 (0%)	1	1
	TNF-α	0/38 (0%)	0/52 (0%)	1	1	0/27 (0%)	0/37 (0%)	1	1
	CD154	0/38 (0%)	0/52 (0%)	1	1	0/27 (0%)	0/37 (0%)	1	1
	IL-4	0/38 (0%)	0/52 (0%)	1	1	0/27 (0%)	0/37 (0%)	1	1
	IL-21	0/38 (0%)	0/52 (0%)	1	1	3/27 (11.11%)	3/37 (8.11%)	0.69	1
	GzB	4/38 (10.53%)	2/52 (3.85%)	0.24	1	21/27 (77.78%)	25/37 (67.57%)	0.41	1
EM	IFN-γ	0/38 (0%)	0/52 (0%)	1	1	0/27 (0%)	0/37 (0%)	1	1
	IL-2	0/38 (0%)	0/52 (0%)	1	1	0/27 (0%)	0/37 (0%)	1	1
	TNF-α	0/38 (0%)	0/52 (0%)	1	1	0/27 (0%)	0/37 (0%)	1	1
	CD154	0/38 (0%)	0/52 (0%)	1	1	3/27 (11.11%)	1/37 (2.7%)	0.3	1
	IL-4	0/38 (0%)	0/52 (0%)	1	1	3/27 (11.11%)	0/37 (0%)	0.07	1
	IL-21	0/38 (0%)	0/52 (0%)	1	1	0/27 (0%)	0/37 (0%)	1	1
	GzB	1/38 (2.63%)	0/52 (0%)	0.42	1	2/27 (7.41%)	5/37 (13.51%)	0.69	1

TD	IFN-γ	0/38 (0%)	0/52 (0%)	1	1	0/27 (0%)	0/37 (0%)	1	1
	IL-2	0/38 (0%)	0/52 (0%)	1	1	0/27 (0%)	0/37 (0%)	1	1
	TNF-α	0/38 (0%)	0/52 (0%)	1	1	0/27 (0%)	0/37 (0%)	1	1
	CD154	0/38 (0%)	0/52 (0%)	1	1	0/27 (0%)	0/37 (0%)	1	1
	IL-4	0/38 (0%)	0/52 (0%)	1	1	0/27 (0%)	0/37 (0%)	1	1
	IL-21	0/38 (0%)	0/52 (0%)	1	1	0/27 (0%)	0/37 (0%)	1	1
	GzB	0/38 (0%)	0/52 (0%)	1	1	1/27 (3.7%)	0/37 (0%)	0.42	1
Naive	IFN-γ	0/38 (0%)	0/52 (0%)	1	1	2/27 (7.41%)	1/37 (2.7%)	0.57	1
	IL-2	0/38 (0%)	0/52 (0%)	1	1	0/27 (0%)	0/37 (0%)	1	1
	TNF-α	0/38 (0%)	0/52 (0%)	1	1	1/27 (3.7%)	2/37 (5.41%)	1	1
	CD154	0/38 (0%)	1/52 (1.92%)	1	1	0/27 (0%)	0/37 (0%)	1	1
	IL-4	0/38 (0%)	0/52 (0%)	1	1	0/27 (0%)	0/37 (0%)	1	1
	IL-21	0/38 (0%)	0/52 (0%)	1	1	6/27 (22.22%)	7/37 (18.92%)	0.76	1
	GzB	7/38 (18.42%)	10/52 (19.23%)	1	1	21/27 (77.78%)	29/37 (78.38%)	1	1

Abbreviations: CM, central memory; EM, effector memory; TD, terminally differentiated
[a] Raw P, original P-value computed based on two-sided Fisher's exact test.
[b] Adj P, P-values adjusted for multiple testing through Benjamini-Hochberg approach.

Supplementary Table 11. Proportion of positive responses in $CD4^-CD8^-$ T cells, NK, NK T and γδT cells at post-vaccination.

			**CSP**				**HBsAg**			
			**Comparator**	**RTS,S/AS01E**	**P value**		**Comparator**	**RTS,S/AS01E**	**P value**	
**Memory subset**	**Functional marker**	**ICS panel**	**Positive/total (%)**	**Positive/total (%)**	**Raw P [a]**	**Adj P [b]**	**Positive/total (%)**	**Positive/total (%)**	**Raw P [a]**	**Adj P [b]**
$CD4^-CD8^-$ T cells	IFN-γ	P1	0/38 (0%)	0/47 (0%)	1	1	2/26 (7.69%)	1/37 (2.7%)	0.56	1
	IL-2	P1	0/38 (0%)	0/47 (0%)	1	1	0/26 (0%)	0/37 (0%)	1	1

	TNF-α	P1	0/38 (0%)	0/47 (0%)	1	1	0/26 (0%)	0/37 (0%)	1	1
	CD154	P1	0/38 (0%)	0/47 (0%)	1	1	2/26 (7.69%)	1/37 (2.7%)	0.56	1
	IL-4	P1	0/38 (0%)	0/47 (0%)	1	1	2/26 (7.69%)	1/37 (2.7%)	0.56	1
	IL-21	P1	0/38 (0%)	0/47 (0%)	1	1	4/26 (15.38%)	5/37 (13.51%)	1	1
	GzB	P1	1/38 (2.63%)	3/47 (6.38%)	0.62	1	15/26 (57.69%)	21/37 (56.76%)	1	1
NK cells	IFN-γ	P1	0/38 (0%)	0/49 (0%)	1	1	0/27 (0%)	0/37 (0%)	1	1
	IL-2	P1	0/38 (0%)	0/49 (0%)	1	1	0/27 (0%)	1/37 (2.7%)	1	1
	TNF-α	P1	0/38 (0%)	0/49 (0%)	1	1	0/27 (0%)	0/37 (0%)	1	1
	CD154	P1	0/38 (0%)	1/49 (2.04%)	1	1	0/27 (0%)	0/37 (0%)	1	1
	IL-4	P1	0/38 (0%)	0/49 (0%)	1	1	0/27 (0%)	0/37 (0%)	1	1
	IL-21	P1	0/38 (0%)	1/49 (2.04%)	1	1	0/27 (0%)	0/37 (0%)	1	1
	GzB	P1	0/38 (0%)	0/49 (0%)	1	1	0/27 (0%)	0/37 (0%)	1	1
NK T-like cells	IFN-γ	P1	0/23 (0%)	0/24 (0%)	1	1	0/12 (0%)	0/16 (0%)	1	1
	IL-2	P1	0/23 (0%)	0/24 (0%)	1	1	0/12 (0%)	0/16 (0%)	1	1
	TNF-α	P1	0/23 (0%)	0/24 (0%)	1	1	0/12 (0%)	3/16 (18.75%)	0.24	1
	CD154	P1	0/23 (0%)	0/24 (0%)	1	1	0/12 (0%)	0/16 (0%)	1	1
	IL-4	P1	0/23 (0%)	0/24 (0%)	1	1	0/12 (0%)	0/16 (0%)	1	1
	IL-21	P1	0/23 (0%)	0/24 (0%)	1	1	2/12 (16.67%)	5/16 (31.25%)	0.66	1
	GzB	P1	1/23 (4.35%)	0/24 (0%)	0.49	1	0/12 (0%)	0/16 (0%)	1	1
NK cells	IFN-γ	P2	0/31 (0%)	0/42 (0%)	1	1	0/23 (0%)	0/28 (0%)	1	1
	IL-2	P2	0/31 (0%)	0/42 (0%)	1	1	0/23 (0%)	0/28 (0%)	1	1
	TNF-α	P2	0/31 (0%)	0/42 (0%)	1	1	0/23 (0%)	0/28 (0%)	1	1
	CD154	P2	0/31 (0%)	0/42 (0%)	1	1	1/23 (4.35%)	3/28 (10.71%)	0.62	1
	IL-4	P2	1/31 (3.23%)	0/42 (0%)	0.42	1	2/23 (8.7%)	1/28 (3.57%)	0.58	1
	GzB	P2	0/31 (0%)	0/42 (0%)	1	1	0/23 (0%)	1/28 (3.57%)	1	1
	IL-10	P2	0/31 (0%)	0/42 (0%)	1	1	4/23 (17.39%)	2/28 (7.14%)	0.39	1
	IL-13	P2	1/31 (3.23%)	0/42 (0%)	0.42	1	1/23 (4.35%)	3/28 (10.71%)	0.62	1
	IL-17	P2	0/31 (0%)	1/42 (2.38%)	1	1	2/23 (8.7%)	3/28 (10.71%)	1	1
γδ T cells	IFN-γ	P2	0/30 (0%)	0/39 (0%)	1	1	4/21 (19.05%)	5/22 (22.73%)	1	1
	IL-2	P2	0/30 (0%)	0/39 (0%)	1	1	0/21 (0%)	1/22 (4.55%)	1	1
	TNF-α	P2	0/30 (0%)	0/39 (0%)	1	1	1/21 (4.76%)	4/22 (18.18%)	0.34	1
	CD154	P2	0/30 (0%)	1/39 (2.56%)	1	1	9/21 (42.86%)	11/22 (50%)	0.76	1
	IL-4	P2	1/30 (3.33%)	0/39 (0%)	0.43	1	0/21 (0%)	0/22 (0%)	1	1

	GzB	P2	0/30 (0%)	0/39 (0%)	1	1	9/21 (42.86%)	8/22 (36.36%)	0.76	1
	IL-10	P2	0/30 (0%)	2/39 (5.13%)	0.5	1	5/21 (23.81%)	0/22 (0%)	0.02	0.57
	IL-13	P2	0/30 (0%)	0/39 (0%)	1	1	6/21 (28.57%)	8/22 (36.36%)	0.75	1
	IL-17	P2	0/30 (0%)	1/39 (2.56%)	1	1	0/21 (0%)	0/22 (0%)	1	1
NK T-like cells	IFN-γ	P2	0/23 (0%)	0/26 (0%)	1	1	0/12 (0%)	0/14 (0%)	1	1
	IL-2	P2	0/23 (0%)	0/26 (0%)	1	1	0/12 (0%)	2/14 (14.29%)	0.48	1
	TNF-α	P2	0/23 (0%)	0/26 (0%)	1	1	0/12 (0%)	0/14 (0%)	1	1
	CD154	P2	0/23 (0%)	2/26 (7.69%)	0.49	1	0/12 (0%)	4/14 (28.57%)	0.1	1
	IL-4	P2	0/23 (0%)	0/26 (0%)	1	1	0/12 (0%)	0/14 (0%)	1	1
	GzB	P2	0/23 (0%)	0/26 (0%)	1	1	0/12 (0%)	0/14 (0%)	1	1
	IL-10	P2	0/23 (0%)	1/26 (3.85%)	1	1	3/12 (25%)	1/14 (7.14%)	0.31	1
	IL-13	P2	0/23 (0%)	1/26 (3.85%)	1	1	3/12 (25%)	3/14 (21.43%)	1	1
	IL-17	P2	0/23 (0%)	0/25 (0%)	1	1	0/11 (0%)	0/14 (0%)	1	1

[a] Raw P, original P-value computed based on two-sided Fisher's exact test.

[b] Adj P, P-values adjusted for multiple testing through Benjamini-Hochberg approach.

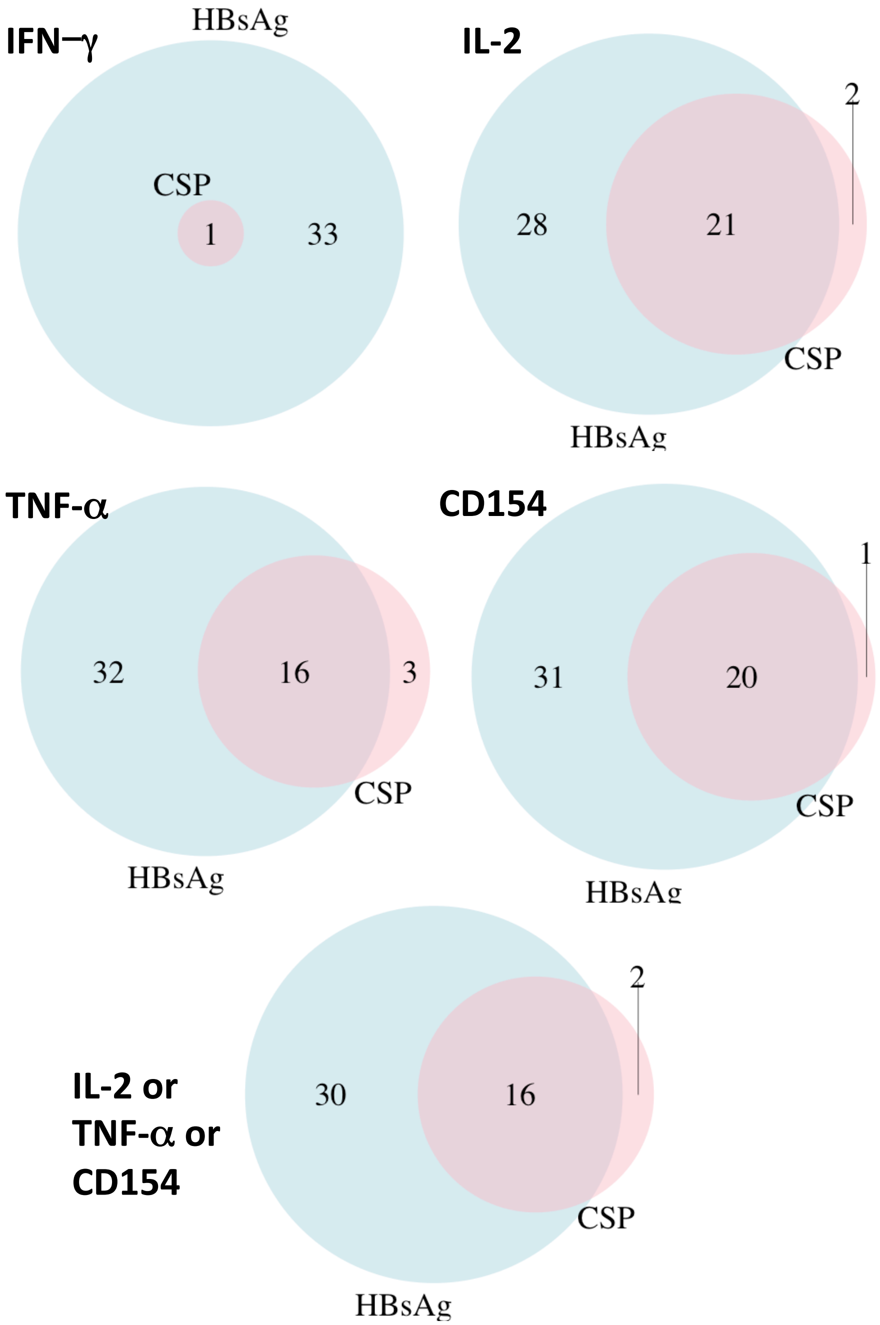

Supplementary Figure 4. Venn diagrams for RTS,S/AS01E vaccinees with CSP and HBsAg positive responses. Venn diagrams show CSP and HBsAg responders from the subset of RTS,S/AS01E-vaccinees that had data from both antigen stimulations. Positivity of responses was determined by MIMOSA. N= 67 RTS,S/AS01E vaccinees.

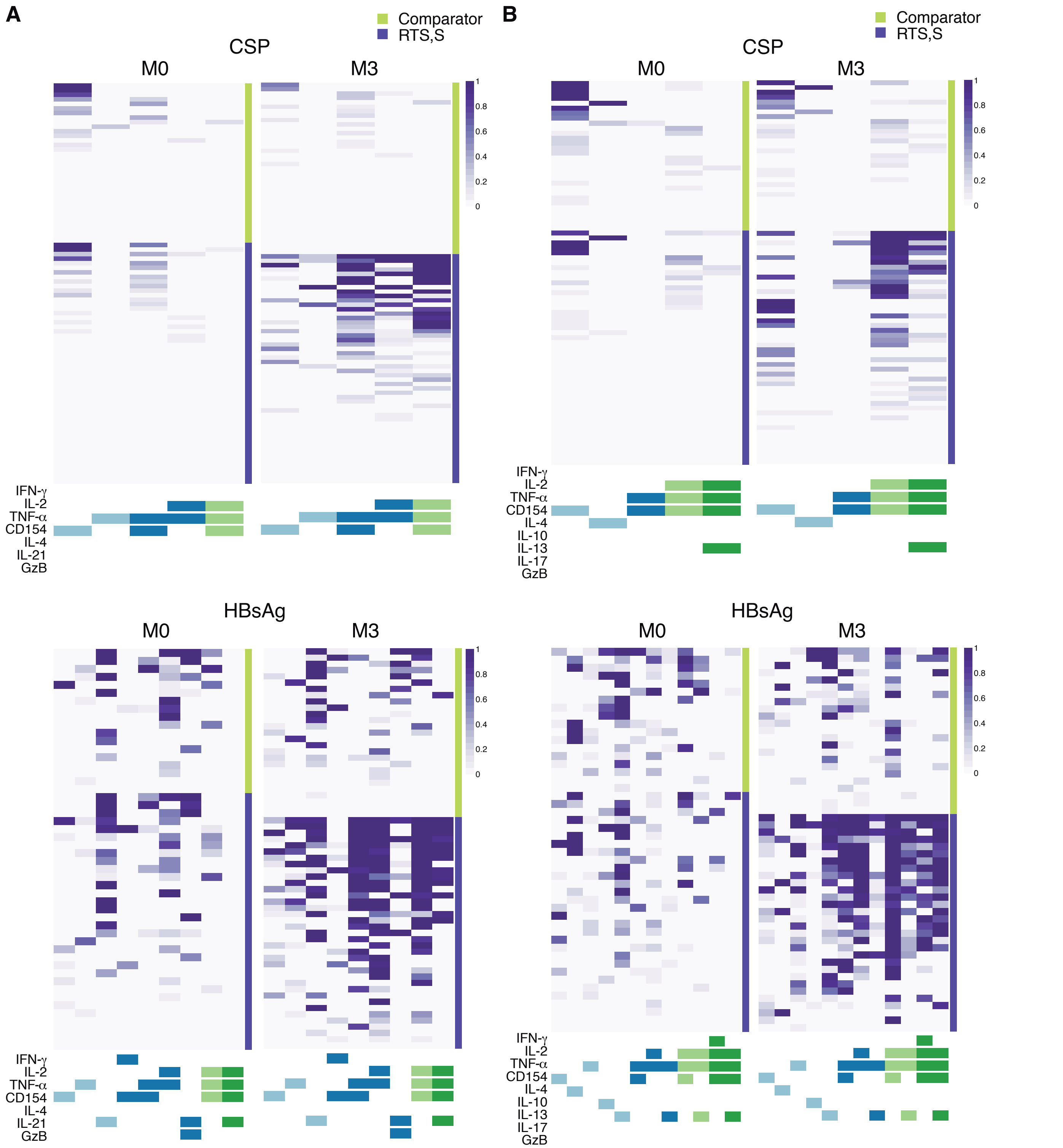

Supplementary Figure 5. Polyfunctional CD4+ T cell responses using the ICS panels P1 (A) and P2 (B). Heatmap of COMPASS posterior probabilities showing CD4+ T cell responses to CSP and HBsAg in RTS,S/AS01E and comparator vaccinees at pre-vaccination (M0) and one month post-third vaccination (M3). Columns represent functional cell subsets with detectable antigen-specific responses, color-coded by the number of functional markers they express and are ordered by

increasing degree of polyfuncitonality. Rows represent study children, which are stratified by vaccine status, at the top the comparator vaccinees and at the bottom the RTS,S/AS01E vaccinees. Each cell shows the probability (color coded by purple intensity) that the corresponding child shows an antigen-specific response in the corresponding cell subset. Sample size in (A) For CSP N= 53 RTS,S/AS01E and 35 comparator at M0, 52 RTS,S/AS01E and 39 comparator at M3; For HBsAg N= 32 RTS,S/AS01E and 18 comparator at M0, 37 RTS,S/AS01E and 27 comparator at M3; (B) For CSP N= 47 RTS,S/AS01E and 30 comparator at M0, 48 RTS,S/AS01E and 31 comparator at M3; For HBsAg N= 30 RTS,S/AS01E and 18 comparator at M0, 30 RTS,S/AS01E and 23 comparator at M3.
